# Spotted phenotypes in horses lost attractiveness in the Middle Ages

**DOI:** 10.1038/srep38548

**Published:** 2016-12-07

**Authors:** Saskia Wutke, Norbert Benecke, Edson Sandoval-Castellanos, Hans-Jürgen Döhle, Susanne Friederich, Javier Gonzalez, Jón Hallsteinn Hallsson, Michael Hofreiter, Lembi Lõugas, Ola Magnell, Arturo Morales-Muniz, Ludovic Orlando, Albína Hulda Pálsdóttir, Monika Reissmann, Matej Ruttkay, Alexandra Trinks, Arne Ludwig

**Affiliations:** 1 Leibniz Institute for Zoo and Wildlife Research, Department of Evolutionary Genetics, 10315 Berlin, Germany; 2 German Archaeological Institute, Department of Natural Sciences, Berlin, 14195 Berlin, Germany; 3 Centro de Ciencias de la Complejidad, Universidad Nacional Autónoma de México, Ciudad de México, Mexico; 4 Landesamt für Denkmalpflege und Archäologie Sachsen-Anhalt – Landesmuseum für Vorgeschichte, 06114 Halle (Saale), Germany; 5 University of Potsdam, Faculty of Mathematics and Natural Sciences, Institute for Biochemistry and Biology, 14476 Potsdam, Germany; 6 The Agricultural University of Iceland, Faculty of Land and Animal Resources, IS-112 Reykjavik, Iceland; 7 Archaeological Research Collection, Tallinn University, Rüütli 10, 10130 Tallinn, Estonia; 8 National Historical Museums, Contract Archaeology, 226 60 Lund, Sweden; 9 Universidad Autonoma de Madrid, Laboratory of Archaeozoology, Madrid, Spain; 10 Centre for GeoGenetics, Natural History Museum of Denmark, University of Copenhagen, 1350K Copenhagen, Denmark; 11 Humboldt University Berlin, Faculty of Life Sciences, Albrecht Daniel Thaer-Institute, 10115 Berlin, Germany; 12 Slovak Academy of Sciences, Institute of Archaeology, 949 21 Nitra, Slovak Republic

## Abstract

Horses have been valued for their diversity of coat colour since prehistoric times; this is especially the case since their domestication in the Caspian steppe in ~3,500 BC. Although we can assume that human preferences were not constant, we have only anecdotal information about how domestic horses were influenced by humans. Our results from genotype analyses show a significant increase in spotted coats in early domestic horses (Copper Age to Iron Age). In contrast, medieval horses carried significantly fewer alleles for these phenotypes, whereas solid phenotypes (i.e., chestnut) became dominant. This shift may have been supported because of (i) pleiotropic disadvantages, (ii) a reduced need to separate domestic horses from their wild counterparts, (iii) a lower religious prestige, or (iv) novel developments in weaponry. These scenarios may have acted alone or in combination. However, the dominance of chestnut is a remarkable feature of the medieval horse population.

The variety of coat colour phenotypes is not only a valued feature of present-day domestic horses but has also fascinated humans ever since prehistoric times. Ancient cave paintings depicted several colour variants of wild horses, and further diversification occurred with domestication. In the early stages of horse domestication, a rapid increase in coat colour variation was enhanced by the artificial selection of horses under human care and their preferences for new phenotypes^1^,^2^.

Today, several horse breeds are named after their coat colours and patterns, reflecting the great interest of humans in horse coloration. However, like most domestic animals, horses have undergone extensive breeding and breed standardization, especially during the last few centuries. Analysing only modern individuals can therefore lead to false conclusions about the history of breeds or the presence/absence of specific traits that may have existed in the past^3^, as has been recently demonstrated in studies on domestic chicken^4^ and horses^5^. Furthermore, human preferences have changed greatly over time and across cultures^6^. Based on our current knowledge, organized animal breeding over a larger region was first implemented in Europe at the time of the Roman Empire^7^. During the Roman period, long-distance exchange of individuals exhibiting favourable phenotypes was conducted to improve local stocks throughout the occupied territories^7^,^8^,^9^. With the fall of the Roman Empire, animal husbandry in Europe became more disorganized and resulted in a wider phenotypic variation of domestic animals^10^,^11^.

The relevance of coat coloration for investigating domestication has been previously demonstrated^6^,^12^,^13^,^14^,^15^. For example, a strong effect of artificial selection on a coat colour locus was discovered in domestic pigs^16^. In horses, new phenotypes emerged soon after the onset of domestication^12^. Subsequently, the alternating frequency of leopard complex spotting in ancient horses suggested changes in human preferences over time^6^,^13^. However, because human preferences have varied over time and because horses started to be differentiated by use during the Middle Ages, a dense temporal and geographical sampling of horses will help establish a better understanding of the history of domestic horse populations. This study addresses the effect of human selection on coat colour phenotypes in horses from the Bronze Age to the Middle Ages, and therefore, has importance for our understanding of breeding preferences and the history of present breeds.

## Results

In this study, we successfully genotyped 107 ancient samples for eight coat colour SNPs. Including data from previous studies^12^,^13^, this dataset of 201 samples (Table S1) displays 14 different phenotypes (Table S4).

**Figure 1:**
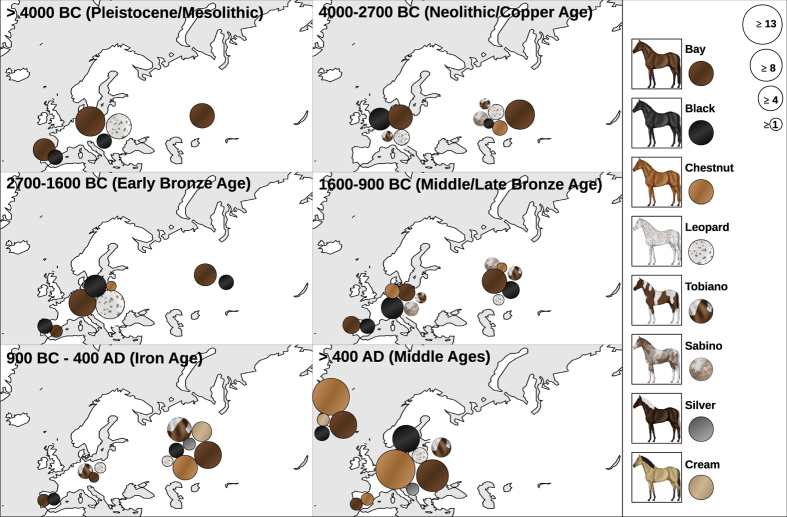
Occurrence of eight horse coat colour phenotypes based on their location for six different periods. For each spotted phenotype (leopard, tobiano and sabino) all samples, independent of their basic colour (bay, black or chestnut), are included. The map was made using Gimp 2.8.10 (www.gimp.org).

Figure 1 provides an overview of the number of different phenotypes for the respective region over six time periods. However, it should be noted that for spotted phenotypes (leopard, tobiano and sabino), all individuals are pooled irrespective of their basic coat colour (bay, black or chestnut). Early domestic horses (4000–2700 BC) showed six phenotypes, of which three were already present in pre-domestic horses (>4000 BC^12^,^13^). In both the Bronze and Iron Ages (2700–900 BC and 900 BC-400 AD, respectively), nine phenotypes were already present (detailed information on phenotype numbers in Table S4).

Two Neolithic samples exhibited the tobiano phenotype, a colour pattern only occurring in domestic horses. One horse was from Salzmünde, Germany (cal. 3368–3101 BC), and the other from Botai, Kazakhstan (cal. 3654–3630 BC). Prior to this study, this phenotype had not been observed in horses before 1500 BC.

The differences in observed allele frequencies at different time periods were significant for different combinations of alleles and periods (see Supplementary Table S5). Alleles associated with new phenotypes, which became conspicuous in the early stages of domestication (chestnut ‘*e*’, tobiano ‘*KM1*’ and sabino ‘*SB1*’), decreased substantially in the Early Bronze Age. However, alleles for sabino and tobiano spotting (*SB1* and *KM1*) in particular increased again in frequency during the Late Bronze and Iron Ages, with *KM1* reaching an observed frequency of approximately 0.19 in the Iron Age (Figures S1–S3). Due to the occurrence of a few leopard alleles at the beginning of the medieval period, the inferred history of allele frequencies suggests that they were not decreasing during that time period. However, when the Middle Ages are separated into early and late periods, the change becomes significant, and the simulations show a strong decrease of spotted alleles driven by negative selection (Figure S5).

**Figure 2:**
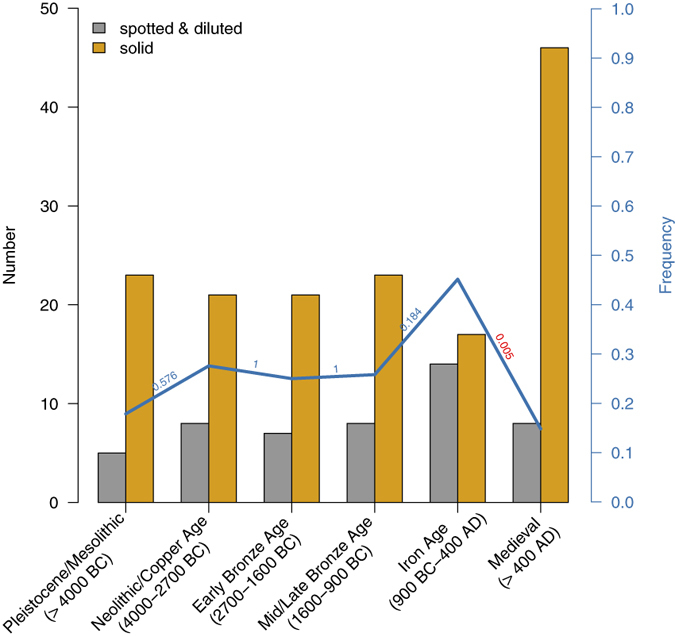
Numbers and frequencies of all spotted/diluted versus basic-coloured (solid) phenotypes for six different periods. The bar plot displays the absolute number of horses with the respective phenotype (grey represents all spotted and diluted horses, yellow represents horses with a solid coat colour). The blue line indicates the frequency of the spotted/diluted coat-colour variants, and the numbers above it indicate the significance of the changes in frequency, which were calculated using Pearson’s chi-squared test.

Most of the newly genotyped samples (N = 107) came from medieval European horses (after 400 AD, N = 55), which displayed a significant drop in the frequency of dilution or spotting phenotypes (Iron Age: 14 out of 31, Middle Ages: 8 out of 56, p = 0.005, based on Pearson’s chi-squared test) but a greater frequency of solid coat colours than horses of pre-Medieval Times (Fig. 2), which can also be observed in the lower allelic frequencies for most of the non-wild type alleles in the sample from the Middle Ages (Figure S1). Three medieval horses carried the pearl allele (*prl*). Since we did not detect this allele in any pre-medieval samples (N = 52), these samples currently represent the oldest detection of this allele. However, as heterozygous carriers, they did not exhibit the respective phenotype and, therefore, are not depicted in Fig. 1.

Although samples are neither chronologically nor geographically homogeneously distributed, there is no indication for any strong regional differences (Table S6). Medieval horses from various locations in Europe displayed similar frequencies of coat colour-associated alleles and their respective phenotypes. Nevertheless, Icelandic horses from the 9^th^–11^th^ century (N = 21) did not harbour any spotted colour patterns despite this being a characteristic of current Icelandic horses.

**Figure 3:**
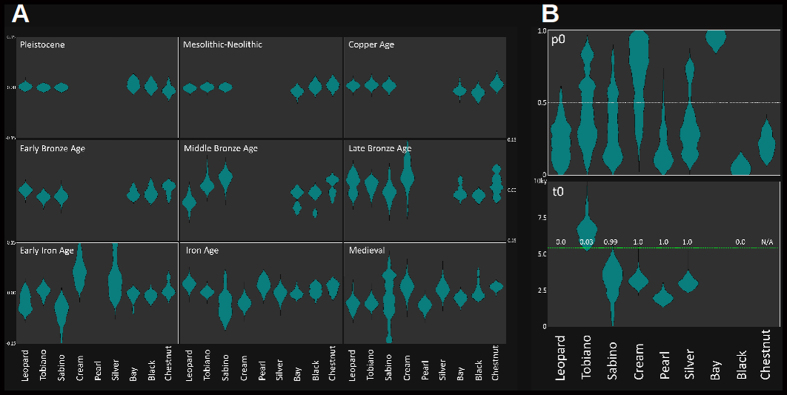
(**A**) Violin plots representing the posterior distributions of the parameters that were inferred by simulation-MCMC. Each chart represents a phenotype and each violin plot a selection coefficient in each period (indicated). The two parameters at right are the initial allele frequency and the age of the derived allele. The empty spaces (e.g. Cream phenotype in the Middle Bronze Age) occur because some simulations had several periods merged due to the derived allele appeared very late in the sampling, making the selection at the earliest periods pointless. (**B**) Violin plots of the initial allele frequency (top) and age (bottom) of the derived alleles. The empty places correspond to the cases when the derived allele first appeared in the Pleistocene, the allele associated to the corresponding phenotype was not derived (Bay); or the introduction of alleles didn’t get an appreciable probability. The green line represents the time of domestication, and the numbers above the probabilities that the age of the derived allele was younger than domestication.

In order to infer selection coefficients and temporal paths of allele frequencies we developed a novel MCMC method for time series of genetic data (see methods and Supplementary Appendix S1). It allowed inferring a striking rise in the frequency of the ‘black’ ASIP allele (‘a’) prior to domestication followed by an equally striking rise of the frequency for the chestnut allele. Figure 3 depicts the selection coefficients for the various phenotypes (basic coat colours in the upper row) as inferred by our method. With domestication, black and chestnut horses increased in frequency at the expense of bay horses, which is reflected in the significantly positive selection coefficients (p < 0.01). This trend was reversed in horses from the late Bronze and Iron Ages when the frequency and selection coefficient of chestnut horses increased, whereas the trend for black and bay phenotypes showed negative selection. Medieval horses again showed a significantly positive selection coefficient for black and chestnut alleles. This alternating pattern in selection coefficients is reflected in the respective allele frequencies for the derived *ASIP* and *MC1R* alleles (‘*a*’ and ‘*e*’, Figure S1), which have shown an apparent pattern of alternation since the onset of domestication (Figure S1).

For the dilution phenotypes, the information carried by the data (only 3–4 alleles were detected) was scarce providing limited information in the inference and showing a rise in Iron Age to Medieval times (Table S1; Figures S1 and S3).

Finally, after we incorporated the possibility that derived alleles (for novel phenotypes) “appear” spontaneously (emulating their new “appearance” by mutation) at some point between the beginning of the simulation and their first detection, we found that simulations in which the alleles newly emerged in the population, showed a much higher probability compared to those in which the allele was forced to exist since the beginning of the simulation. This finding strongly suggests that all derived alleles for diluted and spotted phenotypes appeared via mutation in the Holocene (after domestication), with the exception of the leopard complex spotting (*LP*) allele, which has existed since the Pleistocene (prior to domestication) supporting previous findings in horses and other domestic animals that the age of the alleles coding for colour variants only found in domestic animals tend to be very young when detected and likely occurred after domestication^2^.

## Discussion

Our extended dataset highlights important changes in human preferences for horse phenotypes. Specifically, the large number of samples from the Middle Ages demonstrate significant differences in coat colour phenotypes between pre-medieval and medieval horses. Spotted and diluted horses were considerably more frequent during the Bronze Age and Iron Age, whereas solid phenotypes, especially chestnut, were predominant in the Middle Ages. In addition, we discovered that tobiano spotting, which only occurs in domestic horses and had thus far only been detected after 1500 BC, was present in the Eneolithic/Copper Age (Kazakhstan, cal. 3654–3630 BC, and Germany, cal. 3368–3101 BC). Similar to chestnut and sabino spotting, the tobiano phenotype appears to arise shortly after domestication, which is assumed to have started approximately 4000–3500 BC in the Ponto-Caspian steppe region (modern day Kazakhstan and Ukraine)^17^. That the tobiano allele most likely did not exist before domestication but emerged later is further supported by the estimated “time to the introduction” (age) of the allele. The same was observed for the Gait-keeper mutation in domestic horses, which has a rather recent origin in early medieval England^18^. Moreover, the detection of the tobiano phenotype in two Eneolithic domestic horses from distant regions has important implications regarding the origins of horse domestication^17^ and their subsequent distribution. It also supports previous claims that the emergence of domestic horses in Central Europe at the end of the fourth millennium BC was facilitated by introduced horses from the Ponto-Caspian steppe^19^. The occurrence of the tobiano phenotype and its allelic frequency through time display a similar pattern as that of the leopard (*LP*) complex spotting allele^6^. In the Iron Age, the tobiano allele (‘*KM1*’) occurred even more frequently than the *LP* allele. Both the high allele frequency and the fairly positive selection coefficient indicate that humans favoured spotted horses during the Iron Age. Still, as was the case for horses with the leopard spotting phenotype, tobiano horses were obviously not equally sought after during all time periods given their complete absence in the early and middle Bronze Age and their low prevalence in the early Middle Ages. Although the reason for these varying preferences through time cannot be resolved at this point, pleiotropic disadvantages may have played an important role^1^,^20^,^21^.

In contrast to their Iron Age ancestors, early medieval horses displayed a strong reduction in spotted and diluted phenotypes. Such a decrease had apparently started in Roman times when, according to ancient Roman records, horses with uniform coat colour were preferred to spotted horses, as the latter were considered to be of inferior quality^7^. Historically, the early Middle Ages were marked by the collapse of the Roman Empire and large-scale human migrations across Eurasia and Northern Africa. The Roman Empire represented a cultural and economic entity characterized by, among other things, unprecedented agricultural advances. Its decline caused substantial changes in animal husbandry throughout the former empire, which was also observed in changes of livestock phenotypes^10^,^11^. One might predict that less specialized breeding, with individuals not being as actively selected for desired traits and thus an increased effect of genetic drift, would further facilitate the reduced frequency of spotted coats and colour dilutions in horses. Considering possible pleiotropic disadvantages associated with some spotted and diluted phenotypes^20^,^21^,^22^, especially in homozygous individuals^22^,^23^,^24^, such a reduction might even have been enhanced by negative selection.

Spotted phenotypes might also have served as a visible cue to distinguish domestic from wild horses. With the decline of wild horse populations, the necessity to maintain a marker that set apart domestic animals, which had greater performance and higher economic value, might have been rendered obsolete.

**Figure 4:**
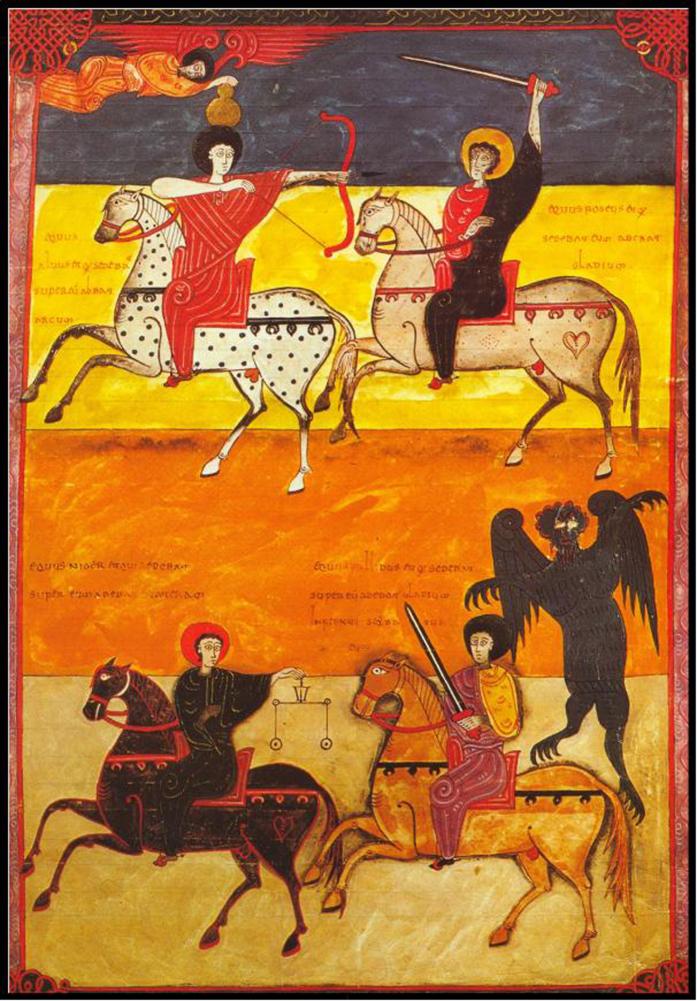
Four Horsemen of the Apocalypse on the *Beato de Fernando I y doña Sancha* dated 1047 AD. (Apoc. VI, 1–8f. 135; shelf 14-2 National Library, Madrid; https://commons.wikimedia.org/wiki/File:B_Facundus_135.jpg).

Additionally, religious symbolism may have played a role in the decline of spotted horses during Medieval times. At the beginning of this period spotted horses appear to have been desirable. In the Apocalypse of St. John dated AD 81–96^25^, where the end of the world revolves around the fall of Rome, the symbolism concerns the coat colour of the horse of each rider: the rider of victory has a white or white spotted horse, the rider of Famine a black horse, the rider of Death a bay horse and the rider of War a chestnut horse (Fig. 4). In other words, only the white/white spotted horse had a positive connotation, and this may explain the preferences of royalty for these horse phenotypes and the praise they received from authors such as Isidore of Seville^26^,^27^. In time, the Church transmogrified the rider of victory into that of the Triumphant Church, a powerful icon of Medieval Christian symbolism^28^. However, by an ironic twist of history, during the second half of the Middle Ages, after a series of epidemics -the Black Death among them- wiped out half of the European population, the single “good” rider was replaced by a fourth “bad” rider, that of the Plague^28^, but the colours of the four horses remained the same resulting in the white or spotted horse now also being ridden by a rider with a negative connotation. Whether that switch meant that the negative connotation of the rider extended to the horse he was depicted on and spotted coats therefore became an undesirable “trait” for horses, which fostered the decline of spotted phenotypes remain open questions at this point, although the results of our study might grant support to this idea.

Notably, our study on ancient domestic horses contains a sampling bias because many medieval samples originate from graves of knights and noblemen. The high frequency of chestnut coat colour among these horses fits well with horse colours of the apocalyptic riders in which the rider of war had a chestnut horse. Moreover, chestnut horses are generally characterized as bold^29^, which was definitely a beneficial behaviour for war horses. However, our samples do not necessarily represent working horses. Likely, the horses of the upper-class were bred for more fashionable traits. In the same manner as white horses were reserved for royalty^2^ noblemen may have also had special preferences that are beyond our present-day understanding. Finally, improvements in weaponry (i.e., the longbow) may have influenced the shift in coat colour preferences. A diluted or spotted horse may simply have been an easier target for enemies to see, especially over long distances or while moving.

We detected the allele for the pearl dilution (*prl*), an equine colour variant caused by a recessive mutation of the *MATP* gene, in an Iberian, a German and a Slovakian sample from the Middle Ages. In present-day horses, this phenotype occurs only in Iberian breeds and breeds that are descended from them (e.g., Peruvian Paso)^30^. Although our results prove the existence of the pearl allele in horses outside the Iberian Peninsula in historic times, two factors argue for drift as a likely cause for its disappearance in non-Iberian horses: (i) the low observed frequency of this allele and (ii) the fact that the phenotype is visible only in homozygous individuals or in horses with an additional cream allele.

Strikingly, the Icelandic horses from the Viking Age show clear differences from their modern counterparts. The samples we analysed originated from graves around Iceland that date back to as early as the middle of the 9^th^ century, shortly after Iceland was settled. These horses already carried the allele for Silver dapple (two of 19 individuals), a common trait in contemporary Icelandic horses, but they did not carry any alleles associated with spotted phenotypes, although such individuals are frequent in modern Icelandic horses^31^,^32^,^33^. Therefore, we assume that spotted phenotypes were introduced after the 10^th^/11^th^ century AD, which contradicts the popular claim that the import of horses to Iceland was prohibited for almost 1000 years^34^.

In conclusion, the present study is the most comprehensive to date addressing coat colour differences in ancient horses. We found differential selection for spotted and solid phenotypes over time reflecting changes in human preferences. With the decline of the Roman Empire, solid phenotypes increased in frequency, whereas the frequency of spotted and diluted phenotypes decreased significantly. Although detailed inferences concerning geographic differences cannot be made, we observed considerable regional changes between medieval and present-day horses.

## Methods

### Ancient DNA analysis, authentication and sequencing

DNA extraction was performed in a specialized trace DNA laboratory at the Leibniz Institute for Zoo and Wildlife Research (Ludwig Laboratory) in Berlin, Germany, following standard procedures to avoid contamination. Independent replications were carried out in the ancient DNA laboratory at the University of Potsdam (Hofreiter Laboratory), Germany. To minimize contamination in all tissue, the surfaces of the samples were removed by abrasion. Next, 100–200 mg of bone or tooth was ground with a cryomill and incubated in 1 ml of an extraction buffer (0.45 M EDTA, 0.25 mg/ml proteinase K, pH 8.0) overnight with rotation at 37 °C. The following steps were conducted according to an optimized extraction protocol for short DNA fragments^35^.

All samples were analysed for eight mutations located in six genes (*MC1R, ASIP, PMEL17, MATP, TRPM1, KIT*), including alleles for the basic coat colours (bay, black, chestnut), dilution phenotypes (cream, silver, pearl) and white spotting (tobiano, sabino, leopard). In addition to the ones described in previous studies^12^,^13^ a SNP in the MATP gene^30^ associated with the Pearl phenotype was included (Table S2). Multiplex PCRs were performed in 20-μl reaction volumes using a 4 μl DNA extract and 1x AmpliTaq Gold PCR buffer II (ABI), 4 mM MgCl2; 1 mg/ml bovine serum albumin (BSA); 250 μM dATP, dCTP and dGTP; 500 μM dUTP, 150 nM of each primer and 2 U of AmpliTaq Gold (ABI). To control for carryover contamination, each sample was treated with 1 U of heat-labile uracil-DNA glycosylase in an initial incubation step of 15 min at 37 °C. Target regions were amplified with target-specific primer pairs that had been tagged with common sequence tags (CS1 and CS2, Fluidigm).

Following multiplex amplification, PCR products were sequenced using a combination of multiplexed Illumina MiSeq sequencing and traditional Sanger capillary electrophoresis. For Illumina sequencing, the amplicons were indexed using a maximum of 384 barcodes (containing Illumina adaptor sequences) in an additional amplification step and then pooled. To remove primer and adaptor dimers and other amplification products, the multiplex PCR products were purified using the AMPure PCR purification system (Agencourt) with a 1.8-fold ratio of SPRI beads relative to the reaction volume. The pooled and purified amplicon libraries were then sequenced on an Illumina MiSeq sequencer using CS1 and CS2 as well as their reverse complements as sequencing primers according to the manufacturer’s instructions. For a few samples, the PCR products were generated with the same target-specific primer pairs and then sequenced on an ABI 3130xl Genetic Analyser using the BigDye^®^ Terminator v3.1 Cycle Sequencing Kit (Thermo Fisher Scientific). In addition to the sequencing analyses, the 11 bp deletion in the *ASIP* gene was genotyped via gel electrophoresis.

### Data Analysis

Traditional Sanger sequences were edited and aligned in Geneious v8.1 (Biomatters). Illumina reads were automatically demultiplexed by the Illumina MiSeq software and further processed by applying the following steps: Illumina sequencing adaptors were cut from the reads using cutadapt^35^; reads were then filtered for quality with Trimmomatic 0.33^36^,^37^; all reads that contained more than 8 bp with a Phred score below 20 were discarded and only reads with a minimum length of 50 bp were retained. The trimmed and filtered paired-end reads were merged into a single read with FLASh^38^ to improve their overall quality. Using Bowtie 2^39^ and samtools 0.1.19^40^, the merged reads were mapped to the reference sequences (accession numbers in Table S2) for the respective PCR products. For each sample and locus, a consensus sequence was generated in Geneious v8.1 (Biomatters) from which the genotype and the resulting coat colour phenotype was deduced. To investigate the changes of the number of spotted and solid phenotypes between the different times Pearson’s chi-squared test was applied.

### Allelic dropout

The probability (P) of a false homozygous individual per locus was calculated after n replicates as follows: P = K(K/2)^n−1^, where K is the observed number of allelic dropouts/false homozygote divided by all heterozygous individuals^41^. We did a minimum of three replications, which reduced the risk of the non-detection of a heterozygous individual to 0.2%.

### Temporal test

We applied a Bayesian test of temporal changes in allele frequencies^42^ in order to evaluate the null hypothesis that the observed changes in allele frequencies could be attributed solely to genetic drift and sampling error. For doing so, it was necessary to group the individuals in samples with a common (average) age (see Figure S2). Despite some bias is introduced due to the averaging of ages when grouped, this test can provide clues regarding the potential presence of selection.

### Selection coefficients

To determine how natural and artificial selection could have changed over time, we divided the sampling timeframe in nine periods (Pleistocene, Mesolithic/Neolithic, Copper Age, Early Bronze Age, Middle Bronze Age, Late Bronze Age, Early Iron Age, Iron Age and Medieval) based on historical criteria; but fusing some periods when sample sizes demanded it. Then, we estimated a selection coefficient for each phenotype and period.

The large number of unknown parameters, together with the epistatic and dependency relationships between genes and phenotypes (Figure S4) precluded the use of previously reported methods for detecting selection (e.g. ref. 43), as well as the use of approximate Bayesian computation (ABC), which failed to provide informative posteriors even after we implemented a Sequential Monte Carlo (SMC) procedure that theoretically improved the efficiency of by a factor of 10^9^.

For that reason, we implemented a novel MCMC algorithm which consisted in the simulation of the temporal path of population allele frequencies under a Wright-Fisher model, followed by the analytic calculation of the likelihood of the empirical sample (which employed the simulated allele frequencies). Then we implemented an inference by MCMC based on the likelihoods of the samples given the simulated population frequencies (see Appendix S1 for details). As a Bayesian Monte Carlo method, our method allows to account for relevant sources of uncertainty in the observed data and other variables. This allowed treating properly the uncertainty associated to the effective population sizes, generation time and the ages of the samples, which uncertainty came from stratigraphic or radiocarbon dating.

The MCMC was run under a Metropolis-Hasting algorithm with a Gibbs sampler that was implemented to optimize the exploration of the large parametric space of selection coefficients. The phenotypes or genes that had epistatic or dependency relationships were run together; otherwise the genes were run independently. For each inference we ran 20 chains, 2.0 × 10^4^ steps each (2.0 × 10^5^ for ASIP-MC1R), with a burnin of 50%. Specifics about the MCMC are shown in Tables S7. Simulations were programed following previously published methods^6^,^44^, and modifications were programed to account for the genes and phenotypes interactions. Following literature, the simulations incorporated a growth in horse population, which began with the onset of domestication^1^,^45^,^46^,^47^,^48^,^49^,^50^. The priors for population sizes, generation time and time to the start of the population growth are shown in Table S7. Ages were sampled from normal or uniform priors to take uncertainties in the radiocarbon or stratigraphic age into account, and four samples from the Late Pleistocene were sampled from exponential priors because of their very large stratigraphic range (11.7–126 ka); and the fact that their genotyping was successfully (because ancient DNA decline is exponential) means that their ages are more likely younger than older.

The simulations implemented two models to deal with the initial state of derived alleles, allowing them to exist since the beginning of the simulated time frame (at a frequency being an estimating parameter) or being introduced by mutation at some point between the beginning of the simulated time frame and its first detection (its age being an estimating parameter). The selection of the right model was performed implicitly by allowing the MCMC to shift among models (see ref. 51 and Appendix S1).

The simulations were programed using the code of the programs employed in refs 6 and 41 as template. The source code is available from the following link: https://www.dropbox.com/sh/uns1gsprj7xbbzo/AAAEr4hfya16P5nejpXVVnUfa?dl=0.

## Additional Information

**How to cite this article**: Wutke, S. *et al*. Spotted phenotypes in horses lost attractiveness in the Middle Ages. *Sci. Rep.* **6**, 38548; doi: 10.1038/srep38548 (2016).

**Publisher's note:** Springer Nature remains neutral with regard to jurisdictional claims in published maps and institutional affiliations.

## Supplementary Material

Supplementary Information
