# Author Correction: Spotted phenotypes in horses lost attractiveness in the Middle Ages

**DOI:** 10.1038/s41598-020-62905-z

**Published:** 2020-04-10

**Authors:** Saskia Wutke, Norbert Benecke, Edson Sandoval-Castellanos, Hans-Jürgen Döhle, Susanne Friederich, Javier Gonzalez, Jón Hallsteinn Hallsson, Michael Hofreiter, Lembi Lõugas, Ola Magnell, Arturo Morales-Muniz, Ludovic Orlando, Albína Hulda Pálsdóttir, Monika Reissmann, Matej Ruttkay, Alexandra Trinks, Arne Ludwig

**Affiliations:** 1 Department of Evolutionary Genetics, Leibniz Institute for Zoo and Wildlife Research, 10315 Berlin, Germany; 2 Department of Natural Sciences, German Archaeological Institute, Berlin, 14195 Berlin, Germany; 3 Centro de Ciencias de la Complejidad, Universidad Nacional Autónoma de México, Ciudad de México, Mexico; 4 Landesamt für Denkmalpflege und Archäologie Sachsen-Anhalt – Landesmuseum für Vorgeschichte, 06114 Halle (Saale), Germany; 5 University of Potsdam, Faculty of Mathematics and Natural Sciences, Institute for Biochemistry and Biology, 14476 Potsdam, Germany; 6 The Agricultural University of Iceland, Faculty of Land and Animal Resources, IS-112 Reykjavik, Iceland; 7 Archaeological Research Collection, Tallinn University, Rüütli 10, 10130 Tallinn, Estonia; 8 National Historical Museums, Contract Archaeology, 226 60, Lund, Sweden; 9 Universidad Autonoma de Madrid, Laboratory of Archaeozoology, Madrid, Spain; 10 Centre for GeoGenetics, Natural History Museum of Denmark, University of Copenhagen, 1350K Copenhagen, Denmark; 11 Humboldt University Berlin, Faculty of Life Sciences, Albrecht Daniel Thaer-Institute, 10115 Berlin, Germany; 12 Slovak Academy of Sciences, Institute of Archaeology, 949 21 Nitra, Slovakia

Correction to: *Scientific Reports* 10.1038/srep38548, published online 07 December 2016

The Acknowledgements section in this Article is incomplete.

“This work was funded by the Deutsche Forschungsgemeinschaſt (LU 852/7–4). We thank Pavel Kosintsev for ancient horse samples from the Caspian Sea region. We thank Dietmar Lieckfeldt and Melanie Pruvost for their assistance as well as Dorina Lenz for helping with the data analyses. We also acknowledge the valuable comments from Dolores Carmen Morales-Muñiz about the Riders of the Apocalypse.”

should read:

“This work was funded by the Deutsche Forschungsgemeinschaſt (LU 852/7–4). We thank Pavel Kosintsev for ancient horse samples from the Caspian Sea region. We thank Dietmar Lieckfeldt and Melanie Pruvost for their assistance as well as Dorina Lenz for helping with the data analyses. We also acknowledge the valuable comments from Dolores Carmen Morales-Muñiz about the Riders of the Apocalypse. This project has received funding from the European Research Council (ERC) under the European Union’s Horizon 2020 research and innovation programme (grant agreement No. 681605).”

