# Supplementary material for: Spotted phenotypes in horses lost attractiveness in the Middle Ages
Source: Sci Rep. 2016 Dec 7;6:38548. doi: 10.1038/srep38548 (PMC5141471; doi:10.1038/srep38548)
Supplement: Supplementary Information [file srep38548-s1.pdf]

Supplementary Information

Spotted phenotypes in horses lost attractiveness in the Middle Ages

Saskia Wutke[1], Norbert Benecke[2], Edson Sandoval-Castellanos[3], Hans-Jürgen Döhle[4], Susanne Friederich[4], Javier Gonzalez[5], Jón Hallsteinn Hallsson[6], Michael Hofreiter[5], Lembi Lõugas[7], Ola Magnell[8], Arturo Morales-Muniz[9], Ludovic Orlando[10], Albína Hulda Pálsdóttir[6], Monika Reissmann[11], Matej Ruttkay[12] , Alexandra Trinks[5] & Arne Ludwig[1]*

[1] Leibniz Institute for Zoo and Wildlife Research, Department of Evolutionary Genetics, 10315 Berlin, Germany, [2] German Archaeological Institute, Department of Natural Sciences, Berlin, 14195 Berlin, Germany, [3] Centro de Ciencias de la Complejidad, Universidad Nacional Autónoma de México, Ciudad de México, Mexico, [4] Landesamt für Denkmalpflege und Archäologie Sachsen-Anhalt – Landesmuseum für Vorgeschichte, 06114 Halle (Saale), Germany, [5] University of Potsdam, Faculty of Mathematics and Natural Sciences, Institute for Biochemistry and Biology, 14476 Potsdam, Germany, [6] The Agricultural University of Iceland, Faculty of Land and Animal Resources, IS-112 Reykjavik, Iceland, [7] Archaeological Research Collection, Tallinn University, Rüütli 10, 10130 Tallinn, Estonia, [8] National Historical Museums, Contract Archaeology, 226 60 Lund, Sweden, [9] Universidad Autonoma de Madrid, Laboratory of Archaeozoology, Madrid, Spain, [10] Centre for GeoGenetics, Natural History Museum of Denmark, University of Copenhagen, 1350K Copenhagen, Denmark, [11] Humboldt University Berlin, Faculty of Life Sciences, Albrecht Daniel Thaer-Institute, 10115 Berlin, Germany, [12] Slovak Academy of Sciences, Institute of Archaeology, 949 21 Nitra, Slovak Republic

* To whom correspondence should be addressed. E-mail: ludwig@izw-berlin.de

Appendix S1

Estimating selection by simulations coupled with Markov Chain Monte Carlo

The model

Our goal is to estimate a set of selection coefficients (nine coefficients in a single gene/single phenotype system) plus the initial allele frequency, or alternately the age of the allele (see below). Other unknown quantities, as effective population sizes (N_e), generation time and time to the start of the growth are relevant for the model but could not be reliably estimated so they can be considered noise variables. The empirical data, constituted by a set of allele frequencies and sample times are typically known quantities but they can also have some uncertainty. For simplicity, we'll start omitting the noise variables. In the Bayesian framework our specific goal is to approach the posterior probability density function (pdf) of the parameters of interest (hence termed θ) conditional to the empirical data (hence termed x):

$$f(\theta|x) = cf(x|\theta)\pi(\theta)$$

where c is a constant and $\pi(\theta)$ is the joint prior of the parameters. The main challenge is to estimate the likelihood, $f(x|\theta)$, because the relationship between x and θ involves an allele frequencies path, P, which is unobserved and high dimensional (infinite-dimensional if the time is continuous, or n-dimensional if the timeframe has n discrete generations)[1,2]. This estimation has to be performed by marginalizing the likelihood by integrating upon all the possible paths;

$$f(x|\theta) = \int_P f\,(x|P)f(P|\theta) \qquad (1)$$

This is problematic because involves solving partial differential equations or finding the joint distribution of allele frequency paths numerically[1]. The strategies that have been employed for addressing this problem include a continuous diffusion approximation[2], a birth-death type Markov chain[3], an analytic solution by the spectral representation of the transition density[4], and a path augmentation method[1].

Our approach sought a solution based on simulations due to the potential of simulations to deal with the complexity provided by the interaction among genes. Our approach exploited the fact that the two multiplicands in equation (1) can be estimated rather easily but in different ways, despite the intricacies of their joint integration. The first term could be calculated analytically because the empirical data consist of a series of binomial draws that are independent, so:

$$f(x|P) = \prod_{i=1}^{m} \binom{n_i}{k_i} p_{t_i}^{k_i} \left(1 - p_{t_i}\right)^{n_i - k_i} \qquad (2)$$

where m is the number of samples, n_i and k_i are the sizes and count of mutant alleles in the i-th sample respectively, and p_{t_i} the population allele frequency in the generation in which the i-th sample was taken. Let's recall that an allele frequencies path is a series of frequencies $P = \{p_0, p_1, \dots, p_{t_i}, \dots, p_{t_m}\}$.

The second term in eq. (1), is the probability of a path given parameters, $f(P|\theta)$, and its integration can be solved using different strategies (e.g. [1-5]), but also explicit simulations. The most notable advantage of the simulations relies in their capacity to accommodate different sources of complexity, not only those related with intra- or inter-gene relationships (e.g. dominance or epistasis) but virtually any source of complexity, as long as it can be computer-simulated.

The novelty of our approach consists in integrating the likelihood $f(x|\theta)$ in a hybrid method that mixes the analytical calculation of $f(x|P)$ by means of (2) and the sampling of allele frequency paths from its distribution, $f(P|\theta)$, by means of simulation. The key point is that it can be done by a MCMC in which chains accept the proposal states on the basis of the incomplete likelihood provided by $f(x|P)$ (calculated with eq. (2)) and the prior probabilities, because the remaining part of the likelihood is implicitly incorporated when the simulation of allele frequency paths are generated according with their probability. Here we have the strong assumption that the algorithm generates the allele frequency paths from $f(P|\theta)$, which is a reasonable assumption in our case (as we employed probed Wright-Fisher algorithms) but it would need assurance in other systems. Furthermore, the coupling with the prior distributions can be done at once by simulating the paths with parameter values that have been sampled from the prior distributions in order to directly obtain a sample from $f(\theta|x)$:

$$f(\theta|x) = \int_P f\,(x|P) f(P|\theta) \pi(\theta)$$

This method showed a notable capacity to deal with the complexities of our inferential problem. In contrast, other available methods can't deal with complicated relationships between genotypes and phenotypes, as they assign selection coefficients directly to the genotypes. From the many methods available to estimate selection from time series of genetic data, only two[3,4] are able to deal with dominance/recessive relationships between alleles but none is able to deal with epistatic relationships among genes. It looks also unlikely that the methods described in [1-5] can deal with uncertainty in samples ages which is relevant because it makes the order of the samples uncertain and with them the observed frequencies in time. Another option for inferring selection from time series of allele frequencies consist in using approximate Bayesian computation. It has the same (if not a larger) potential to deal with all the interactions and uncertainties, but it is computationally more demanding and probed incapable for the inference of our system due to the number of parameters.

Noise variables

The simulated part of our model can incorporate necessary parameters for which there is neither available knowledge nor sufficient power in the data to estimate them. So the procedures include them as well as their priors and marginalize the target posterior by integrating over their ranges. The parameters that we treated in this way were the effective population sizes and the generation time:

$$f(\theta|x) = \iiint_{PN_eg} f(x|P)f(P|\theta, N_e, g)\pi(\theta, N_e, g)\, \partial P \partial N_e \partial g$$

The simulations sample their values from their prior distributions before inserting them in the simulations. It is important to notice that for each new noise variable, the number of simulations should be increased in the same way as if the number of parameters were increased.

Algorithm

We implemented this algorithm in our problem of estimating selection coefficients for nine periods in a sample of 201 horses spread between the Late Pleistocene and medieval times. The algorithm has the next steps:

1. Define the proper parameters, priors and noise variables. Also define, a model and create a program for the simulation of paths of allele frequencies.
2. Sample a set of parameters θ_0 from their prior distribution $\pi(\theta)$.
3. Simulate an allele frequency path, P_i, using Wright-Fisher explicit simulations with the desired level of complexity and employing the values of θ_i obtained in the previous step.
4. Calculate analytically the partial likelihood of the i-th simulation $L_i = f(x|P_i)\pi(\theta_i)$.
5. Use an appropriate kernel for proposing a new set θ_{i+1}.
6. Simulate an allele frequency path, P_{i+1}, using Wright-Fisher explicit simulations with the desired level of complexity and employing the values of θ_{i+1} obtained in the previous step.
7. Calculate analytically the partial likelihood $L_{i+1} = f(x|P_{i+1})\pi(\theta_{i+1})$.
8. Accept or reject $\{P_{i+1}, \theta_{i+1}\}$ with probability $L_{i+1} \cdot L_i^{-1}$ (or with a Metropolis-Hasting or another criterion).
9. Go to 2.
10. Repeat 1-9 for a sufficiently large number of steps.
11. Run several chains and evaluate different transition kernels. Evaluate and optimize as a regular MCMC procedure.

The algorithm is also able to incorporate some of the many refinements developed under the theory of MCMC. One of them, a Gibbs sampler could be particularly useful for highly multivariate problems. In our algorithm the Gibbs sampler updated the chains one parameter at a time.

Initial states and introduction of alleles

The different methods that have been designed for estimating selection by means of time series of allele frequencies have dealt with the initial state of a derived allele (the one under selection) in two alternate ways: (*i*) the derived allele exist since the beginning of the timeframe of interest, requiring the estimation of its initial allele frequency; or (*ii*) considering that the mutant allele must have appeared by mutation at some time, the estimation of the initial allele frequency is substituted by the estimation of age of the allele.

Considering that our simulations are, in general, more computationally demanding than other approaches (e.g. analytic solutions), our method will generally be circumscribed to the sampling timeframe in order to minimize computational demands. For that reason, the default option will be (*i*). However, if the mutant allele is absent from the first sample there could be a non-null probability that the allele appeared by mutation inside the timeframe of the simulations. Opting for (*i*) or (*ii*) could be solved by performing a Bayesian model comparison between those models. However, it is possible to implement a MCMC that can jump between models in the same way that it jumps between parametric states. This has been done in phylogenetic inference for choosing models of molecular evolution on-the-go[6].

In our case, the oldest sampling of the derived allele was always much younger than the oldest sample, requiring this hybrid approach.

Programing

We created three versions of the algorithm: one for one-gene-systems, one for basic colours (programing the interactions between the genes ASIP and MC1R) and one with all the eight genes and their interactions. Our program consisted in:

1. Reading data and operators for the MCMC;
2. Performing the simulations, each one consisting in:
    a. Nested cycles for: aborting ill-conceived simulations (e.g. when alleles got prematurely extinct); cycling for parameters optimization (Gibbs sampler); and cycling for simulation of each gene's path;
    b. Sampling ages and values of parameters from priors or jumps of the MCMC chain. Update of the likelihood;
    c. Likelihood of the initial sample and posterior samples (inside the generations cycling);
    d. Cycling of generations, including sections for introduction of alleles, definition of new selection coefficients at the corresponding generations, and update of the likelihoods (concerning selection coefficients);
    e. Change of generation from binomial sampling coupled with adjustments for selection. The later ones involved calculation of allele frequencies, genotypic frequencies, augmentation by selection, sampling of the next generation and re-calculation of allele frequencies;
    f. Instructions for the MCMC (acceptance-rejection of new state and update of the chain).
3. Organization and saving of results.

The algorithm was programed in Microsoft Visual Studio 2015 and the Intel Parallel Studio 2013, employing the language Fortran 90. The specifics of the sampling and changes of generation followed[7,8].

Bottom line

Our method has several innovative steps but in its general form is analogue to the estimation of

demographic parameters by coalescent approaches[9]. It has been successfully tested in a simpler system, and showed more accurate than approximate Bayesian computation while being significantly more efficient (see Figure S7 & [10]). Such features could make this technique a useful complement of available methods not only for inferring selection but in other types of inference, especially in those in which complexity precludes the use of other approaches.

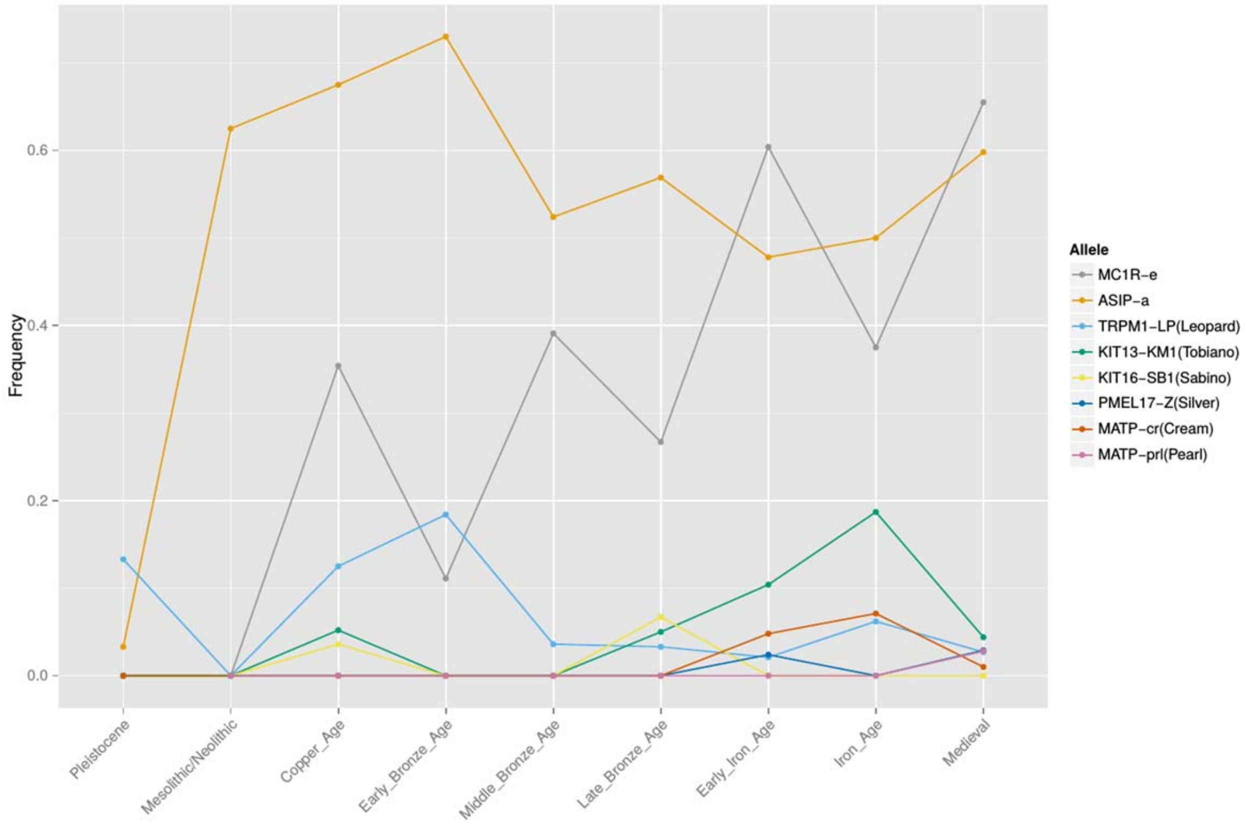

Figure S1: Allele frequencies over time of derived, non-wild-type alleles that are associated with a specific coat color phenotype.

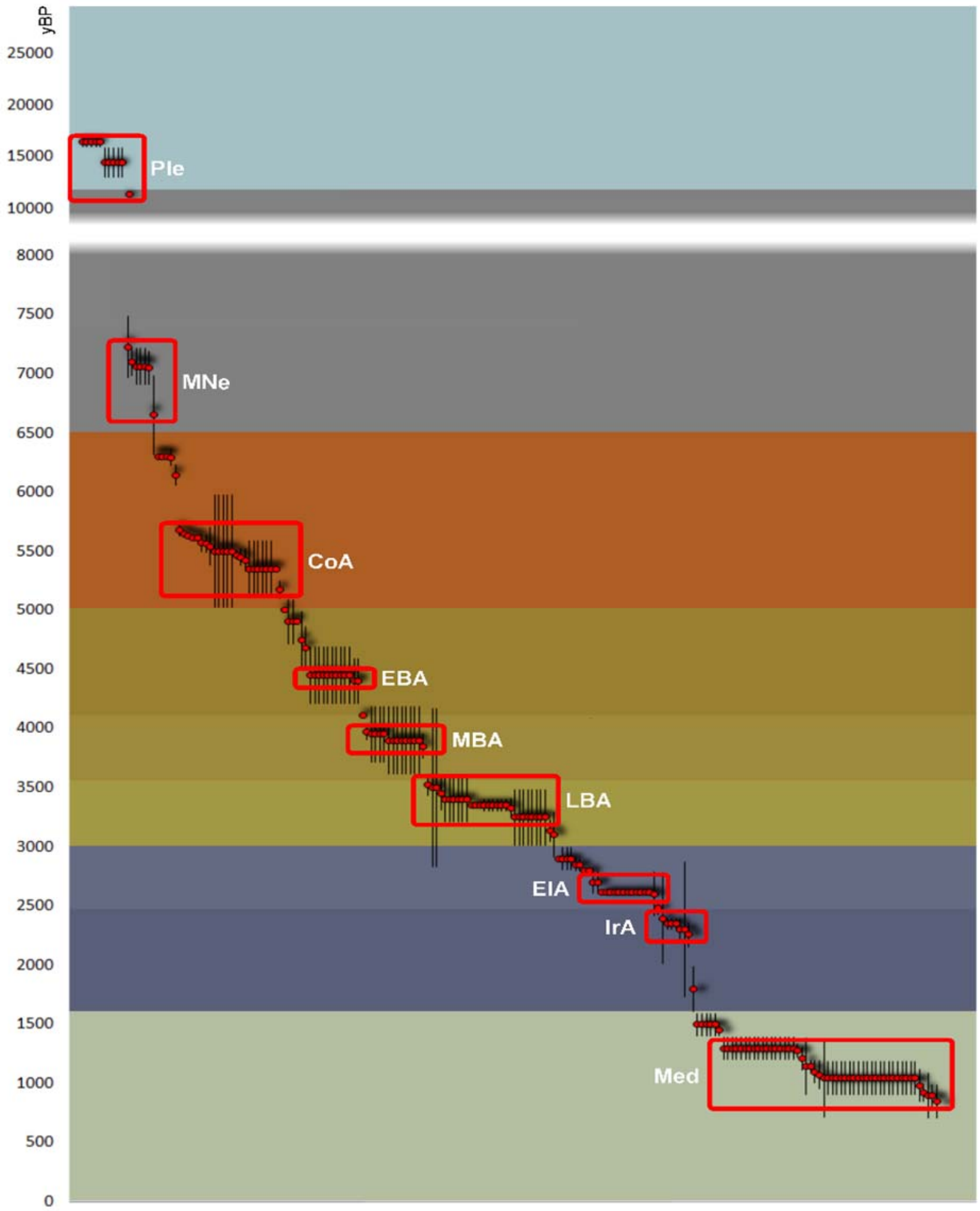

Figure S2. Grouping of samples for the temporal test of allele frequencies. Only the samples inside boxes were included in the test. Notice that the temporal scale changes and is discontinuous for the Pleistocene.

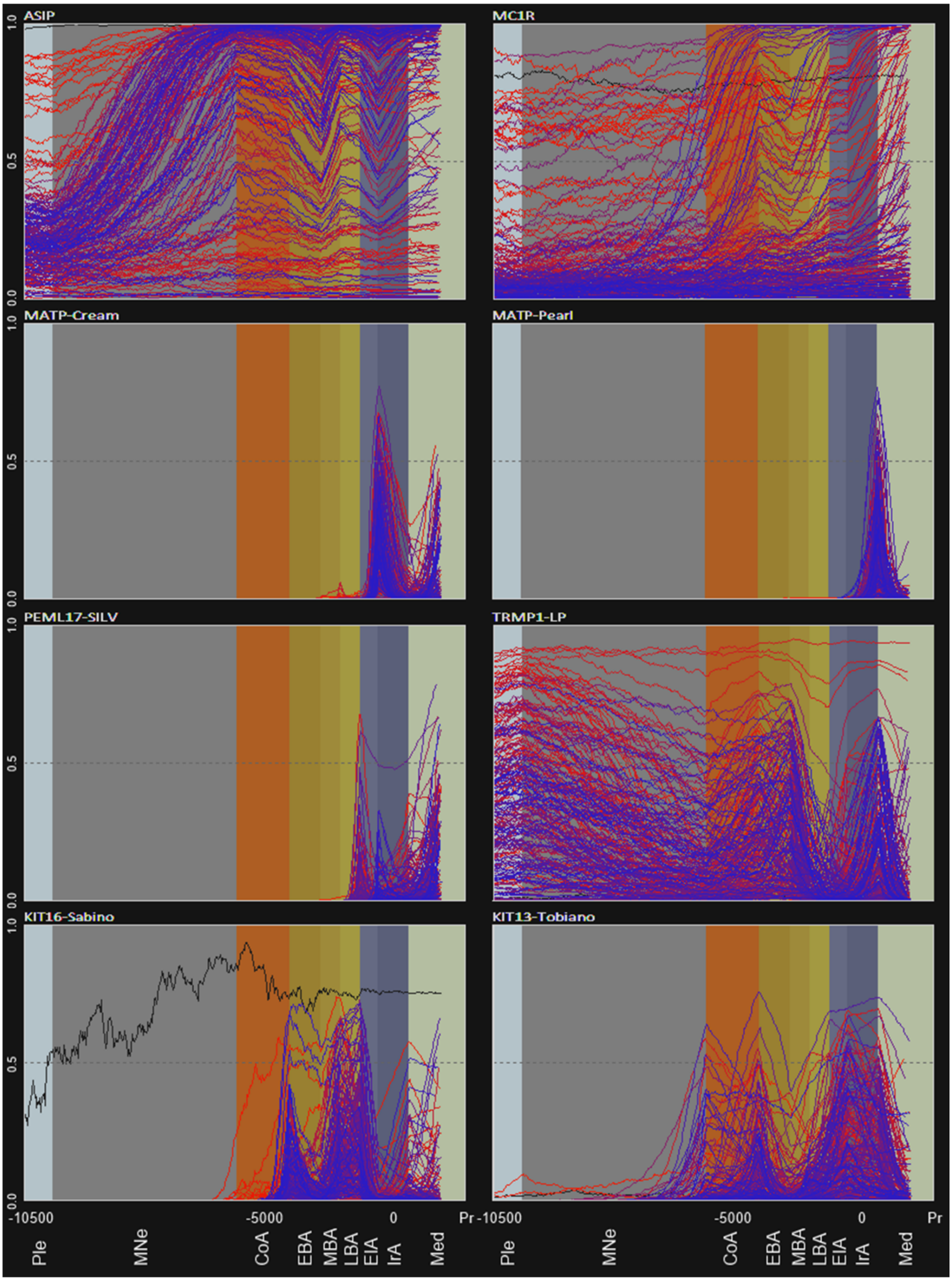

Figure S3: Time trajectories of the allele frequencies in eight genes involved in horse coat color. The frequencies correspond to the derived allele. The codes for the nine time periods indicated at the bottom and with different colors in the background correspond to: Ple=Pleistocene; MNe=Mesolithic-Neolithic; CoA=Copper Age; EBA=Early Bronze Age; MBE=Middle Bronze Age; LBA=Late Bronze Age; EIA=Early Iron Age; IrA=Iron Age; and Med=Medieval (see details in the main text). The time frame ranges from 12.5 ky BP to present (from left to right); most of Pleistocene was omitted for visualization purposes. Each line corresponds to one simulation and the color black corresponds to the initial simulation, the red to simulations of the burnin stage of the MCMC, and blue to simulations employed for the inference.

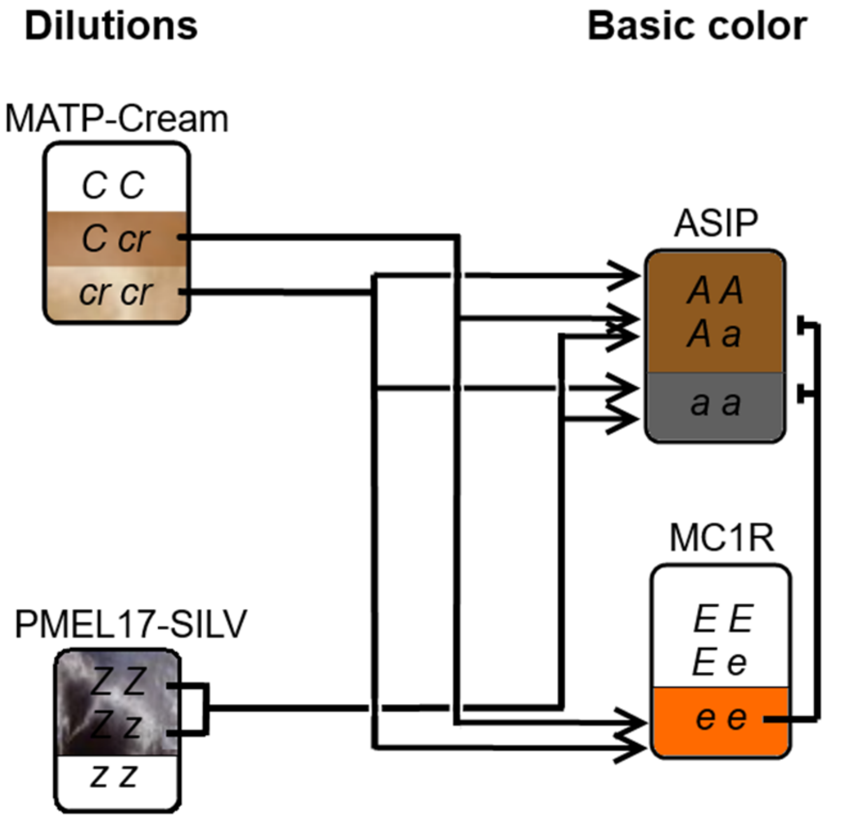

Figure S4. Relationships among phenotypes and genotypes associated to two dilution genes and two basic colors genes. The arrows mean that a dilution gene can only be expressed in the specific color the arrow are directed to (notice that, for instance, cream heterozygotes, *C/cr*, cannot be expressed in black horses, ASIP *a/a*). The t-shape ending of the lines mean that the indicated phenotypes are dominant-repressed by the genotype they come from. The genes that doesn't appear in the figure, KIT13-Tobiano, KIT16-Sabino, MATP-Pearl and TRPM1-LP present phenotypes equivalent regardless the basic color and only present relationships of dominance between alleles of the same gene.

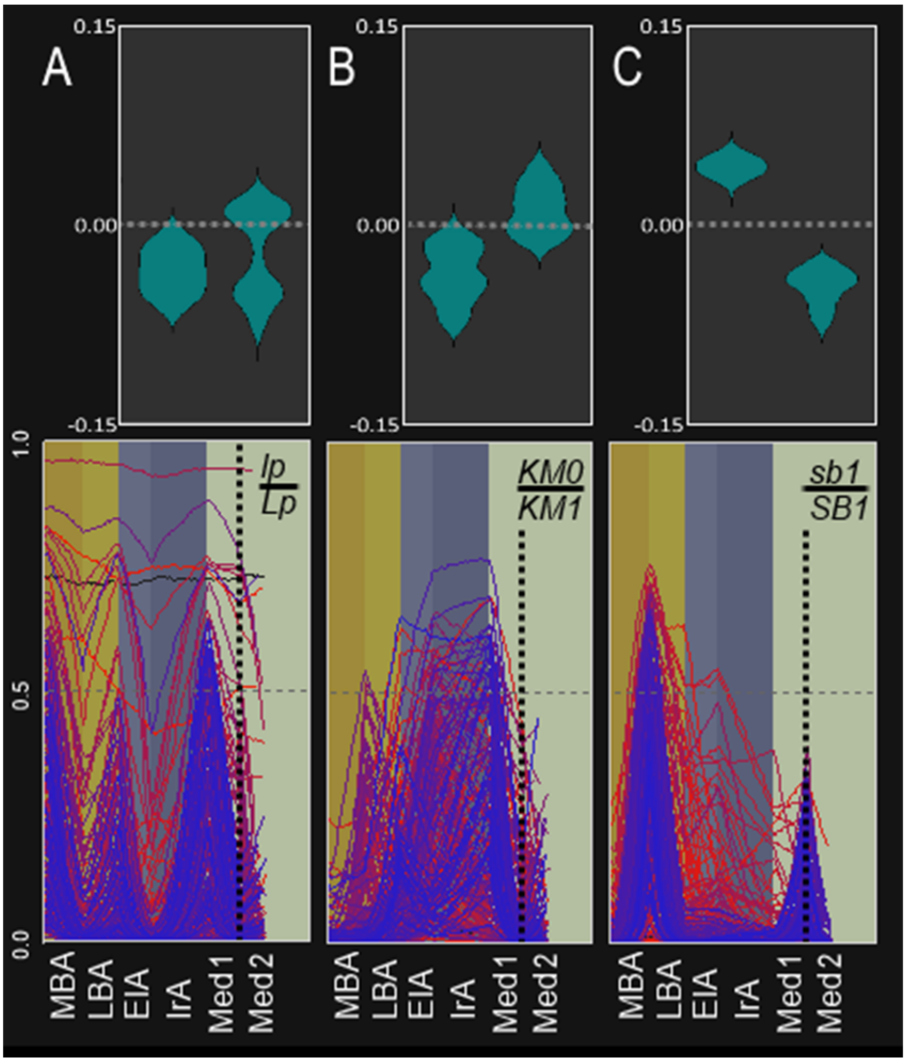

Figure S5. Violin plots of selection coefficients and paths of allele frequencies when the Medieval period was split. The upper panels represent the violin plots of the selection coefficients in the periods Med1 and Med2 (early and late Medieval respectively). The lower panels show the path of allele frequencies; the vertical dotted line divides the Med1 and Med2 periods. A, B, C are for the loci TRPM1-LP (leopard), KIT13-Tobiano and KIT16-Sabino respectively. Unexpectedly, the split not always translated in the later medieval period having a negative selection coefficient (as in C), but in all cases the result was a strong depletion of the derived (spotting) allele due to negative selection.

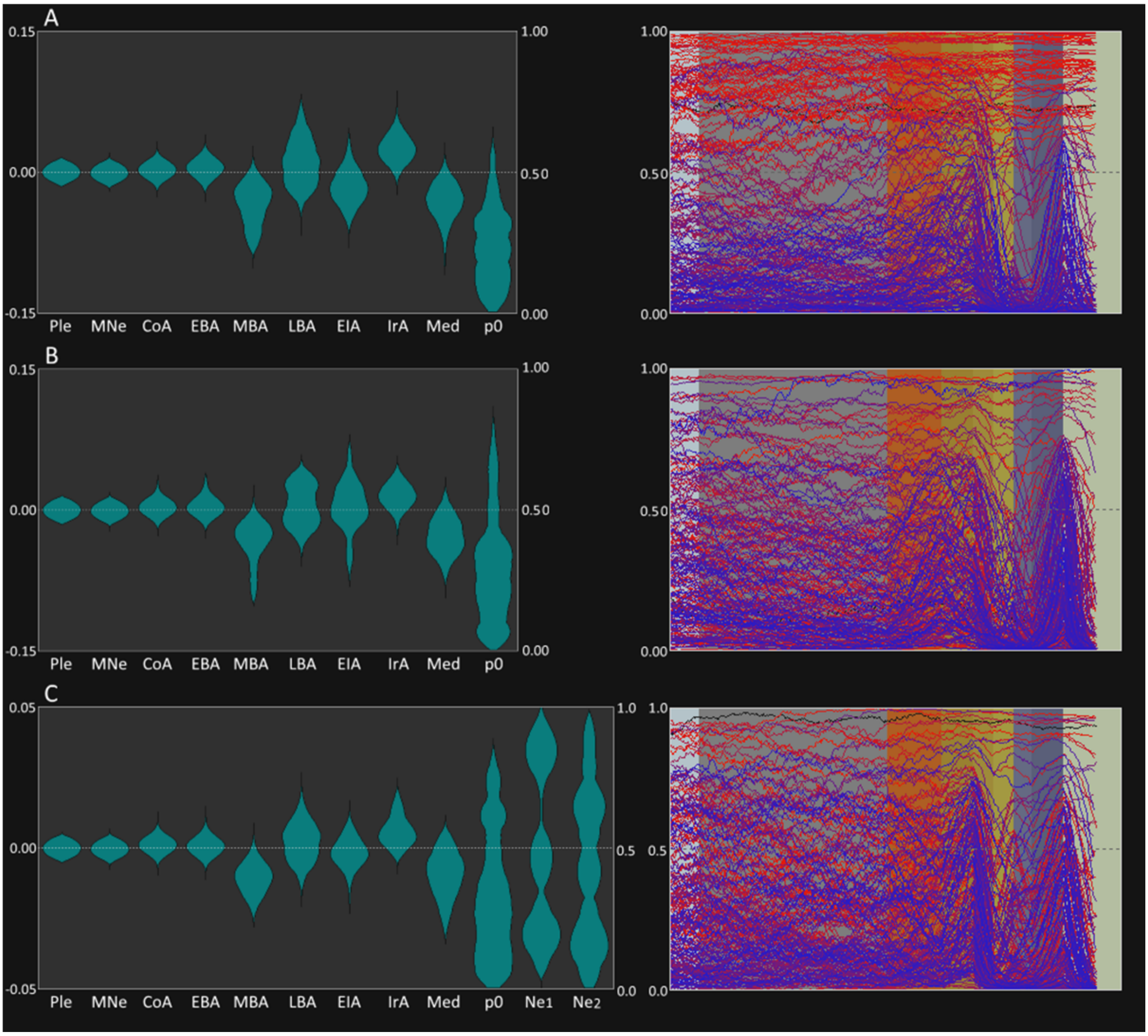

Figure S6. Violin plots of the posterior distributions of parameters (left) and allele frequencies paths (right) as inferred by three types of analyses. (A) The analysis considering a demographic growth starting around the time of the onset of domestication; (B) the analysis with a constant effective population size from the Pleistocene to the Medieval period; (C) the analyses in which g, $Ne1$, $Ne2$ and the time of the start of the growth were treated as parameters instead of noise variables.

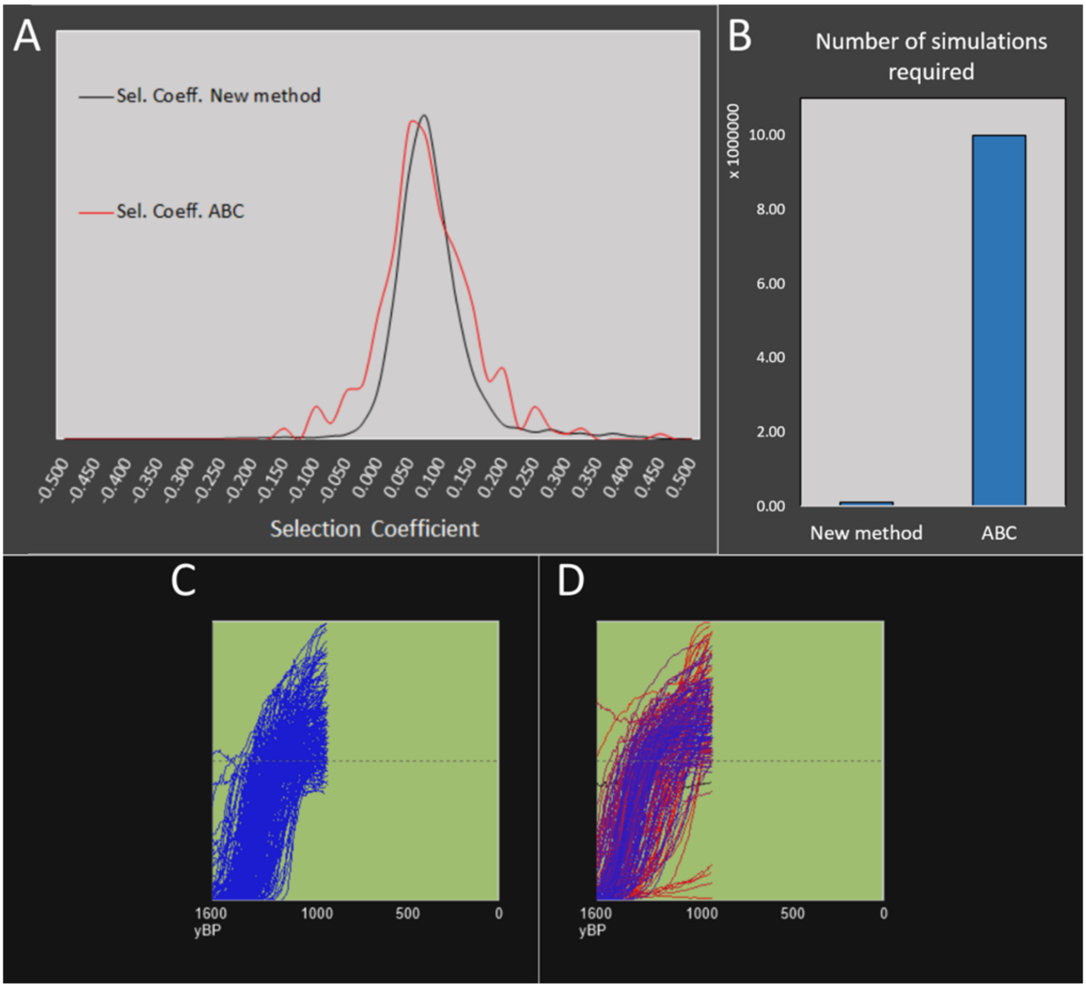

Figure S7. Comparison between the simulation-MCMC and approximate Bayesian computation (ABC). This figure was modified from [10] in a study with many similarities of our own, including the analysis, by means of ancient DNA, of a derived mutation presumably favored by artificial selection in horses. However, the analysis only attained a single period resulting in a much lower dimensionality. (A) Shows the posterior probability distribution of the selection coefficient with ABC and with the simulation-MCMC. (B) shows the number of simulations required for the inference. (C) and (D) show the inferred allele frequency paths obtained by ABC and the simulation-MCMC respectively. The colors in (D) represent the progression of the MCMC showing the initial steps of the chain in red and the latest in blue.

Table S1: Sample information and genotyping results for all successfully typed horses**.**

		Sample	Age		Location	Excavation	Wild/ Domestic	Phenotype			Genotype								
			Stratigraphic date	14C date				Basic	Pattern	Dilution	*ASIP*	*EDNRB*	*KIT13-Tobiano*	*KIT16-Sabino*	*MATP-Cream*	*MATP-Pearl*	*MC1R*	*PMEL17-SILV*	*TRPM1-LP*
Plesitocene/Neolithic/Copper_Age	Asia	**SP1181A**	39.460±400BP		Siberia	Maliy Lyakhovsky Isl.	W	Bay	-	-	*A/A*	*ov/ov*	*KM0/KM0*	*sb1/sb1*	*C/C*	*N/A*	*E/E*	*z/z*	*lp/lp*
		**SP1181B**	Late Pleistocene		Siberia	Bol'shoy Lyakhovsky Isl.	W	Bay	-	-	*A/A*	*ov/ov*	*KM0/KM0*	*sb1/sb1*	*C/C*	*N/A*	*E/E*	*z/z*	*lp/lp*
		**SP1181C**	Late Pleistocene		Siberia	Bol'shoy Lyakhovsky Isl.	W	Bay	-	-	*A/A*	*ov/ov*	*KM0/KM0*	*sb1/sb1*	*C/C*	*N/A*	*E/E*	*z/z*	*lp/lp*
		**SP1181E**	Late Pleistocene		Siberia	Oyagosskiy Yar, Kondrat'evo R., mouth	W	Bay	-	-	*A/A*	*ov/ov*	*KM0/KM0*	*sb1/sb1*	*C/C*	*N/A*	*E/E*	*z/z*	*lp/lp*
		**SP1181F**	Late Pleistocene		Siberia	Kotel'niy Isl., Anisiy Cape	W	Bay	-	-	*A/A*	*ov/ov*	*KM0/KM0*	*sb1/sb1*	*C/C*	*N/A*	*E/E*	*z/z*	*lp/lp*
		**874-9**	4000-3000 BC		Siberia	Krasnokamenka	D	Bay	-	-	*A/A*	*ov/ov*	*KM0/KM0*	*sb1/sb1*	*C/C*	*N/A*	*E/e*	*z/z*	*lp/lp*
		**874-4**	4000-3000 BC		Siberia	Krasnokamenka	D	Bay	Sabino + Leopard	-	*A/A*	*ov/ov*	*KM0/KM0*	*SB1/sb1*	*C/C*	*N/A*	*E/e*	*z/z*	*LP/lp*
		**876-8**	4000-3000 BC		Siberia	Krasnokamenka	D	Bay	-	-	*A/a*	*ov/ov*	*KM0/KM0*	*sb1/sb1*	*C/C*	*N/A*	*E/e*	*z/z*	*lp/lp*
		**874-11**	4000-3000 BC		Siberia	Krasnokamenka	D	Chestnut	-	-	*a/a*	*ov/ov*	*KM0/KM0*	*sb1/sb1*	*C/C*	*N/A*	*e/e*	*z/z*	*lp/lp*
		**874-6**	4000-3000 BC		Siberia	Krasnokamenka	D	Bay	Sabino + Leopard	-	*A/A*	*ov/ov*	*KM0/KM0*	*SB1/sb1*	*C/C*	*N/A*	*E/e*	*z/z*	*LP/lp*
		**715-625**	3600-3000 BC		Kazakhstan	Botai	D	Bay	-	-	*A*	*ov/ov*	*KM0/KM0*	*sb1/sb1*	*C/C*	*N/A*	*E*	*z/z*	*lp/lp*
		**715-660**		cal. 3503-3428 BC	Kazakhstan	Botai	D	Bay	-	-	*A*	*ov/ov*	*KM0/KM0*	*sb1/sb1*	*C/C*	*N/A*	*E*	*z/z*	*lp/lp*
		**715-700**	3600-3000 BC		Kazakhstan	Botai	D	Bay	-	-	*A*	*ov/ov*	*KM0/KM0*	*sb1/sb1*	*C/C*	*N/A*	*E*	*z/z*	*lp/lp*
		**715-1487**	3600-3000 BC		Kazakhstan	Botai	D	Bay	-	-	*A*	*ov/ov*	*KM0/KM0*	*sb1/sb1*	*C/C*	*N/A*	*E*	*z/z*	*lp/lp*
		**715-1547**	3600-3000 BC		Kazakhstan	Botai	D	Bay	-	-	*A*	*ov/ov*	*KM0/KM0*	*sb1/sb1*	*C/C*	*N/A*	*E*	*z/z*	*lp/lp*
		**715-2514**		cal. 3621-3610 BC	Kazakhstan	Botai	D	Bay	Leopard	-	*A*	*ov/ov*	*KM0/KM0*	*sb1/sb1*	*C/C*	*N/A*	*E*	*z/z*	*LP/lp*
		**715-2661**	3600-3000 BC		Kazakhstan	Botai	D	Bay	-	-	*A*	*ov/ov*	*KM0/KM0*	*sb1/sb1*	*C/C*	*N/A*	*E*	*z/z*	*lp/lp*
		**715-494**		cal. 3620-3611 BC	Kazakhstan	Botai	D	Bay	Leopard	-	*A*	*ov/ov*	*KM0/KM0*	*sb1/sb1*	*C/C*	*N/A*	*E*	*z/z*	*LP/lp*
		**715-705**		cal. 3654-3630 BC	Kazakhstan	Botai	D	Bay	Tobiano	-	*A*	*ov/ov*	*KM1/KM0*	*sb1/sb1*	*C/C*	*N/A*	*E*	*z/z*	*lp/lp*
		**715-1496**		cal. 3644-3619 BC	Kazakhstan	Botai	D	Bay	-	-	*A*	*ov/ov*	*KM0/KM0*	*sb1/sb1*	*C/C*	*N/A*	*E*	*z/z*	*lp/lp*
		**1427-59**		cal. 2920-2878 BC	Siberia	Alexandrovskoe IV	D	Black	-	-	*a/a*	*ov/ov*	*KM0/KM0*	*sb1/sb1*	*C/C*	*N/A*	*E/E*	*z/z*	*lp/lp*
		**BER1**	3000 BC		Siberia	Denisova-Pescera	D	Chestnut	-	-	*A/A*	*ov/ov*	*KM0/KM0*	*sb1/sb1*	*C/C*	*N/A*	*e/e*	*z/z*	*lp/lp*
	Europe/Asia(minor)	**PET6**	14000-11000 BC		Germany	Petersfels	W	Bay	Leopard	-	*A/A*	*ov/ov*	*KM0/KM0*	*sb1/sb1*	*C/C*	*N/A*	*E/E*	*z/z*	*LP/lp*
		**PET5**	14000-11000 BC		Germany	Petersfels	W	Bay	Leopard	-	*A/a*	*ov/ov*	*KM0/KM0*	*sb1/sb1*	*C/C*	*N/A*	*E/E*	*z/z*	*LP/lp*
		**PET3**	14000-11000 BC		Germany	Petersfels	W	Bay	-	-	*A/A*	*ov/ov*	*KM0/KM0*	*sb1/sb1*	*C/C*	*N/A*	*E/E*	*z/z*	*lp/lp*
		**PET2**	14000-11000 BC		Germany	Petersfels	W	Bay	-	-	*A/A*	*ov/ov*	*KM0/KM0*	*sb1/sb1*	*C/C*	*N/A*	*E/E*	*z/z*	*lp/lp*
		**PET1**	14000-11000 BC		Germany	Petersfels	W	Bay	Leopard	-	*A/A*	*ov/ov*	*KM0/KM0*	*sb1/sb1*	*C/C*	*N/A*	*E/E*	*z/z*	*LP/lp*
		**KG5**	15000-14000 BC		Germany	Kniegrotte	W	Bay	Leopard	-	*A/A*	*ov/ov*	*KM0/KM0*	*sb1/sb1*	*C/C*	*N/A*	*E/E*	*z/z*	*LP/lp*
		**KG4**	15000-14000 BC		Germany	Kniegrotte	W	Bay	-	-	*A/A*	*ov/ov*	*KM0/KM0*	*sb1/sb1*	*C/C*	*N/A*	*E/E*	*z/z*	*lp/lp*
		**KG3**	15000-14000 BC		Germany	Kniegrotte	W	Bay	-	-	*A/A*	*ov/ov*	*KM0/KM0*	*sb1/sb1*	*C/C*	*N/A*	*E/E*	*z/z*	*lp/lp*
		**KG2**	15000-14000 BC		Germany	Kniegrotte	W	Bay	-	-	*A/A*	*ov/ov*	*KM0/KM0*	*sb1/sb1*	*C/C*	*N/A*	*E/E*	*z/z*	*lp/lp*
		**KG1**	15000-14000 BC		Germany	Kniegrotte	W	Bay	-	-	*A/A*	*ov/ov*	*KM0/KM0*	*sb1/sb1*	*C/C*	*N/A*	*E/E*	*z/z*	*lp/lp*
		**Pie05**	4300 BC		Romania	Pietrele	W	Bay OR Black	-	-	*N/A*	*ov/ov*	*KM0/KM0*	*sb1/sb1*	*C/C*	*PRL/PRL*	*E/E*	*z/z*	*lp/lp*
		**Pie06**	4300 BC		Romania	Pietrele	W	Bay OR Black	Leopard	-	*N/A*	*ov/ov*	*KM0/KM0*	*sb1/sb1*	*C/C*	*PRL/PRL*	*E/E*	*z/z*	*LP/lp*
		**PIE9**	4300 BC		Romania	Pietrele	W	Black	-	-	*a/a*	*ov/ov*	*KM0/KM0*	*sb1/sb1*	*C/C*	*N/A*	*E/e*	*z/z*	*lp/lp*
		**Spa1**		cal. 9390-9210 BC	Ukraine	Span-Koba	W	Bay	-	-	*A/A*	*ov/ov*	*KM0/KM0*	*sb1/sb1*	*C/C*	*N/A*	*E/E*	*z/z*	*lp/lp*
		**VIT4**		cal. 4360-4220 BC	Romania	Vitanesti	W	Black	-	-	*a/a*	*ov/ov*	*KM0/KM0*	*sb1/sb1*	*C/C*	*N/A*	*E/E*	*z/z*	*lp/lp*
		**Zeg2**		5354 – 5285 BC	Poland	Żegotki 5	W	Bay	-	-	*A/a*	*ov/ov*	*KM0/KM0*	*sb1/sb1*	*C/C*	*N/A*	*E/E*	*z/z*	*N/A*
		**GRO11**	3100-2700 BC		Germany	Großobringen	D	Black	-	-	*a/a*	*ov/ov*	*KM0/KM0*	*sb1/sb1*	*C/C*	*N/A*	*E*	*z/z*	*lp/lp*
		**Salz1**		cal. 3368-3101 BC	Germany	Salzmünde, SK	D	Bay OR Black	Tobiano	-	*N/A*	*ov/ov*	*KM1/KM1*	*sb1/sb1*	*C/C*	*N/A*	*E/E*	*z/z*	*lp/lp*
		**Wes1**	3100-2700 BC		Germany	Westerhausen, HK	D	Bay	-	-	*A/a*	*ov/ov*	*KM0/KM0*	*sb1/sb1*	*C/C*	*PRL/PRL*	*E/E*	*z/z*	*lp/lp*
		**Wes2**	3100-2700 BC		Germany	Westerhausen, HK	D	Bay	-	-	*A/a*	*ov/ov*	*KM0/KM0*	*sb1/sb1*	*C/C*	*PRL/PRL*	*E/E*	*z/z*	*lp/lp*
		**TiS2**	3000-2500 BC		Hungary	Tiszalúc-Sarkad	W	Bay OR Black	-	-	*N/A*	*ov/ov*	*KM0/KM0*	*sb1/sb1*	*C/C*	*PRL/PRL*	*E/E*	*z/z*	*lp/lp*
		**CAS1**		cal. 3700-3380 BC	Romania	Cascioarele	W	Black	-	-	*a/a*	*ov/ov*	*KM0/KM0*	*sb1/sb1*	*C/C*	*N/A*	*E/E*	*z/z*	*lp/lp*
		**May1**	3600-3100 BC		Ukraine	Mayaki	W	Bay OR Black	-	-	*N/A*	*N/A*	*KM0/KM0*	*N/A*	*N/A*	*PRL/PRL*	*E/E*	*z/z*	*lp/lp*
		**MAY3**		cal. 3640-3490 BC	Ukraine	Mayaki	W	Bay	-	-	*A/a*	*ov/ov*	*KM0/KM0*	*sb1/sb1*	*C/C*	*N/A*	*E/E*	*z/z*	*lp/lp*
		**MAY5**		cal. 3250-3100 BC	Ukraine	Mayaki	W	Bay	-	-	*A/a*	*ov/ov*	*KM0/KM0*	*sb1/sb1*	*C/C*	*N/A*	*E/E*	*z/z*	*lp/lp*
		**MAY6**		cal. 3520-3330 BC	Ukraine	Mayaki	W	Bay	-	-	*A/a*	*ov/ov*	*KM0/KM0*	*sb1/sb1*	*C/C*	*N/A*	*E/E*	*z/z*	*lp/lp*
		**MAY7**		cal. 3520-3380 BC	Ukraine	Mayaki	W	Bay	Leopard	-	*A/a*	*ov/ov*	*KM0/KM0*	*sb1/sb1*	*C/C*	*N/A*	*E/E*	*z/z*	*LP/lp*
		**MAY10**		cal. 3650-3500 BC	Ukraine	Mayaki	W	Bay	Leopard	-	*A/a*	*ov/ov*	*KM0/KM0*	*sb1/sb1*	*C/C*	*N/A*	*E/E*	*z/z*	*LP/lp*
		**MOL5**	3720-3630 BC		Ukraine	Molyukhov Bugor	W	Black	-	-	*a/a*	*ov/ov*	*KM0/KM0*	*sb1/sb1*	*C/C*	*N/A*	*E/E*	*z/z*	*lp/lp*
	Iberia	**44**	5500-4950 BC		Spain	Atxoste	W	Bay	-	-	*A/a*	*ov/ov*	*KM0/KM0*	*sb1/sb1*	*C/C*	*N/A*	*E/E*	*z/z*	*lp/lp*
		**3**	5200-4900 BC		Spain	Cueva Fosca -Valencia-Cartellon	W	Black	-	-	*a/a*	*ov/ov*	*KM0/KM0*	*sb1/sb1*	*C/C*	*N/A*	*E/E*	*z/z*	*lp/lp*
		**31**		cal. 5210-4910 BC	Spain	Cueva Fosca -Valencia-Cartellon	W	Bay	-	-	*A/a*	*ov/ov*	*KM0/KM0*	*sb1/sb1*	*C/C*	*N/A*	*E/E*	*z/z*	*lp/lp*
		**32**		cal. 5220-4980 BC	Spain	Cueva Fosca -Valencia-Cartellon	W	Black	-	-	*a/a*	*ov/ov*	*KM0/KM0*	*sb1/sb1*	*C/C*	*N/A*	*E/E*	*z/z*	*lp/lp*
		**34**	5220-4900 BC		Spain	Cueva Fosca -Valencia-Cartellon	W	Bay	-	-	*a/a*	*ov/ov*	*KM0/KM0*	*sb1/sb1*	*C/C*	*N/A*	*E/E*	*z/z*	*lp/lp*
		**37**		cal. 5210-4910 BC	Spain	Cueva Fosca -Valencia-Cartellon	W	Bay	-	-	*A/a*	*ov/ov*	*KM0/KM0*	*sb1/sb1*	*C/C*	*N/A*	*E/E*	*z/z*	*lp/lp*

Table S1: continued.

		Sample	Age	Location	Excavation	Wild/ Domestic	Phenotype			Genotype									
Bronze_Age_early	Asia	270-71	cal. 2031-1905 BC	Siberia	Kulivchi III	D	Black	-	-	a/a	ov/ov	KM0/KM0	sb1/sb1	C/C	N/A	E	z/z	lp/lp
		270-88	2200-1700 BC	Siberia	Kulivchi III	D	Bay	-	-	A	ov/ov	KM0/KM0	sb1/sb1	C/C	N/A	E	z/z	lp/lp
		838-42	2200-1700 BC	Siberia	Sintashta	D	Bay	-	-	A	ov/ov	KM0/KM0	sb1/sb1	C/C	N/A	E	z/z	lp/lp
		838-45	2200-1700 BC	Siberia	Sintashta	D	Bay	-	-	A	ov/ov	KM0/KM0	sb1/sb1	C/C	N/A	E	z/z	lp/lp
		838-64	cal. 2121-2095 BC	Siberia	Sintashta	D	Bay	-	-	A	ov/ov	KM0/KM0	sb1/sb1	C/C	N/A	E	z/z	lp/lp
		838-66	2200-1700 BC	Siberia	Sintashta	D	Black	-	-	a/a	ov/ov	KM0/KM0	sb1/sb1	C/C	N/A	E	z/z	lp/lp
	Europe/Asia(minor)	KAN21	2700-2200 BC	Turkey	Kirklareli-Kanligecit	D	Black	Leopard	-	a/a	ov/ov	KM0/KM0	sb1/sb1	C/C	N/A	E/E	z/z	LP/lp
		KAN22	2700-2200 BC	Turkey	Kirklareli-Kanligecit	D	Black	-	-	a/a	ov/ov	KM0/KM0	sb1/sb1	C/C	N/A	E/E	z/z	lp/lp
		KAN26	2700-2200 BC	Turkey	Kirklareli-Kanligecit	D	Bay	Leopard	-	A/a	ov/ov	KM0/KM0	sb1/sb1	C/C	N/A	E/E	z/z	LP/lp
		KAN27	2700-2200 BC	Turkey	Kirklareli-Kanligecit	D	Black	Leopard	-	a/a	ov/ov	KM0/KM0	sb1/sb1	C/C	N/A	E/E	z/z	LP/lp
		KAN28	2700-2200 BC	Turkey	Kirklareli-Kanligecit	D	Bay	Leopard	-	a/a	ov/ov	KM0/KM0	sb1/sb1	C/C	N/A	E/E	z/z	LP/LP
		KAN29	2700-2200 BC	Turkey	Kirklareli-Kanligecit	D	Black	Leopard	-	a/a	ov/ov	KM0/KM0	sb1/sb1	C/C	N/A	E/E	z/z	LP/lp
		KAN30	2700-2200 BC	Turkey	Kirklareli-Kanligecit	D	Bay	-	-	A/A	ov/ov	KM0/KM0	sb1/sb1	C/C	N/A	E/E	z/z	lp/lp
		KAN31	2700-2200 BC	Turkey	Kirklareli-Kanligecit	D	Black	-	-	a/a	ov/ov	KM0/KM0	sb1/sb1	C/C	N/A	E/E	z/z	lp/lp
		KAN32	2700-2200 BC	Turkey	Kirklareli-Kanligecit	D	Bay	Leopard	-	A/A	ov/ov	KM0/KM0	sb1/sb1	C/C	N/A	E/E	z/z	LP/lp
		KAN33	2700-2200 BC	Turkey	Kirklareli-Kanligecit	D	Black	-	-	a/a	ov/ov	KM0/KM0	sb1/sb1	C/C	N/A	E/E	z/z	lp/lp
		Lor1	cal. 1950-1750 BC	Georgia	Lori-Berd	D	Bay	-	-	A/A	ov/ov	KM0/KM0	sb1/sb1	C/C	N/A	E/E	z/z	lp/lp
		Svi6	2200-1600 BC	Germany	Schloßvippach (Fpl. 51)	D	Bay	-	-	A/a	ov/ov	KM0/KM0	sb1/sb1	C/C	N/A	E/E	z/z	lp/lp
		Alb2	2200-1600 BC	Hungary	Albertfalva	D	Bay	Leopard	-	A/a	ov/ov	KM0/KM0	sb1/sb1	C/C	N/A	E/e	z/z	LP/lp
		TiD2	2200-1600 BC	Hungary	Tiszalúc-Dankadomb	D	Bay OR Black	-	-	N/A	ov/ov	KM0/KM0	sb1/sb1	C/C	PRL/PRL	E/E	z/z	lp/lp
		Bru4	2200-1600 BC	Poland	Bruszczewo Fpl. 5	D	Bay	-	-	A/a	ov/ov	KM0/KM0	sb1/sb1	C/C	PRL/PRL	E/E	z/z	lp/lp
		MKo1	2200-1600 BC	Slovakia	Malé Kosihy	D	Chestnut	-	-	A/A	ov/ov	KM0/KM0	sb1/sb1	C/C	N/A	e/e	z/z	lp/lp
		MKo2	2200-1600 BC	Slovakia	Malé Kosihy	D	Bay	-	-	A/A	ov/ov	KM0/KM0	sb1/sb1	C/C	PRL/PRL	E/E	z/z	lp/lp
		NHr2	2200-1600 BC	Slovakia	Nitriansky Hrádok	D	Bay OR Black	-	-	N/A	ov/ov	KM0/KM0	sb1/sb1	C/C	N/A	E/E	z/z	lp/lp
		Vra2	2200-1600 BC	Slovakia	Vrable	D	Bay	-	-	A/a	ov/ov	KM0/KM0	sb1/sb1	C/C	PRL/PRL	E/e	z/z	lp/lp
	Iberia	CdY2	cal. 2861-2496 BC	Spain	Camino de las Yesereas	D	Black	-	-	a/a	N/A	KM0/KM0	sb1/sb1	C/C	PRL/PRL	E/E	z/z	lp/lp
		Zam09	2600-2200 BC	Portugal	Zambujal	D	Bay	-	-	A/A	ov/ov	KM0/KM0	sb1/sb1	C/C	PRL/PRL	E/E	z/z	lp/lp
		Zam10	2600-2200 BC	Portugal	Zambujal	D	Black	-	-	a/a	ov/ov	KM0/KM0	sb1/sb1	C/C	PRL/PRL	E/E	z/z	lp/lp
Bronze_Age_mid-late	Asia	TAR1	1600-1200 BC	Siberia	Tartas1	D	Bay	-	-	A/a	ov/ov	KM0/KM0	sb1/sb1	C/C	N/A	E/E	z/z	lp/lp
		TAR2	Cal. 1609-1436 BC	Siberia	Tartas1	D	Chestnut	-	-	a/a	ov/ov	KM0/KM0	sb1/sb1	C/C	N/A	e/e	z/z	lp/lp
		TAR4	1600-1200 BC	Siberia	Tartas1	D	Bay	-	-	A/A	ov/ov	KM0/KM0	sb1/sb1	N/A	N/A	E/E	z/z	lp/lp
		TAR5	1600-1200 BC	Siberia	Tartas1	D	Bay	Sabino	-	A/A	ov/ov	KM0/KM0	SB1/sb1	C/C	N/A	E/e	z/z	lp/lp
		TAR7	1600-1200 BC	Siberia	Tartas1	D	Black	-	-	a/a	ov/ov	KM0/KM0	sb1/sb1	C/C	N/A	E/E	z/z	lp/lp
		TAR8	1600-1200 BC	Siberia	Tartas1	D	Bay	-	-	A/a	ov/ov	KM0/KM0	sb1/sb1	C/C	N/A	E/E	z/z	lp/lp
		TAR10	1600-1200 BC	Siberia	Tartas1	D	Black	-	-	a/a	ov/ov	KM0/KM0	sb1/sb1	C/C	N/A	E/E	z/z	lp/lp
		TAR11	cal. 1231-1044 BC	Siberia	Tartas1	D	Black	-	-	a/a	ov/ov	KM0/KM0	sb1/sb1	C/C	N/A	E/e	z/z	lp/lp
		CIC1	1400-1300 BC	Siberia	Cica	W	Bay	-	-	A/A	ov/ov	KM0/KM0	sb1/sb1	C/C	N/A	E/E	z/z	lp/lp
		CIC2	1400-1300 BC	Siberia	Cica	W	Bay	Leopard	-	A/A	ov/ov	KM0/KM0	sb1/sb1	C/C	N/A	E/E	z/z	LP/lp
		CIC3	1400-1300 BC	Siberia	Cica	W	Bay	-	-	A/A	ov/ov	KM0/KM0	sb1/sb1	C/C	N/A	E/E	z/z	lp/lp
		CIC4	1400-1300 BC	Siberia	Cica	D	Chestnut	Tobiano	-	A/a	ov/ov	KM1/KM0	sb1/sb1	C/C	N/A	e/e	z/z	lp/lp
		CIC6	1400-1300 BC	Siberia	Cica	D	Chestnut	Tobiano	-	A/a	ov/ov	KM1/KM0	sb1/sb1	C/C	N/A	e/e	z/z	lp/lp
		CIC8	1400-1300 BC	Siberia	Cica	D	Bay	Sabino	-	A/A	ov/ov	KM0/KM0	SB1/sb1	C/C	N/A	E/E	z/z	lp/lp
	Europe/Asia(minor)	Shi1	cal. 895-795 BC	Armenia	Shirakavan	D	Chestnut	-	-	A/A	ov/ov	KM0/KM0	sb1/sb1	C/C	N/A	e/e	z/z	lp/lp
		Mic1	1500-1000 BC	Moldova	Miciurin	D	Bay	Tobiano	-	A/a	ov/ov	KM1/KM0	sb1/sb1	C/C	N/A	E/E	z/z	lp/lp
		Mic2	1500-1000 BC	Moldova	Miciurin	D	Chestnut	-	-	A/a	ov/ov	KM0/KM0	sb1/sb1	C/C	N/A	e/e	z/z	lp/lp
		Mic3	1500-1000 BC	Moldova	Miciurin	D	Black	-	-	a/a	ov/ov	KM0/KM0	sb1/sb1	C/C	N/A	E/E	z/z	lp/lp
		Mic4	1500-1000 BC	Moldova	Miciurin	D	Bay	-	-	A/a	ov/ov	KM0/KM0	sb1/sb1	C/C	N/A	E/E	z/z	lp/lp
		Mic5	1500-1000 BC	Moldova	Miciurin	D	Bay	Sabino	-	A/a	ov/ov	KM0/KM0	SB1/sb1	C/C	N/A	E/e	z/z	lp/lp
		Gar2	1500-1000 BC	Romania	Garbovat	D	Black	-	-	a/a	ov/ov	KM0/KM0	sb1/sb1	C/C	N/A	E/e	z/z	lp/lp
		Gar3	1500-1000 BC	Romania	Garbovat	D	Bay	-	-	A/a	ov/ov	KM0/KM0	sb1/sb1	C/C	N/A	E/E	z/z	lp/lp
		Gar4	1500-1000 BC	Romania	Garbovat	D	Bay	-	-	A/a	ov/ov	KM0/KM0	sb1/sb1	C/C	N/A	E/E	z/z	lp/lp
		Lch-1	cal. 1410-1250 BC	Armenia	Lchashen	D	Chestnut	Sabino	-	N/A	ov/ov	KM0/KM0	SB1/sb1	C/C	N/A	e/e	z/z	lp/lp
		DuK2	1600-1300 BC	Hungary	Dunaújváros-Koszider	D	Black	-	-	a/a	ov/ov	KM0/KM0	sb1/sb1	C/C	PRL/PRL	E/E	z/z	lp/lp
		TP4	1300-900 BC	Georgia	Tachti Perda	D	Black	-	-	a/a	ov/ov	KM0/KM0	sb1/sb1	N/A	PRL/PRL	E/e	z/z	lp/lp
	Iberia	39	2200-800 BC	Spain	El Acequion	D	Bay	-	-	A/a	ov/ov	KM0/KM0	sb1/sb1	C/C	N/A	E/E	z/z	lp/lp
		40	2200-800 BC	Spain	El Acequion	D	Bay	-	-	A/a	ov/ov	KM0/KM0	sb1/sb1	C/C	N/A	E/E	z/z	lp/lp
		22	1350 BC	Spain	Cueva Rubia-Valmayor/Madrid	D	Black	-	-	a/a	ov/ov	KM0/KM0	sb1/sb1	N/A	N/A	E/E	z/z	lp/lp
		24	1350 BC	Spain	Cueva Rubia-Valmayor/Madrid	D	Bay	-	-	A/a	ov/ov	KM0/KM0	sb1/sb1	N/A	N/A	E/e	z/z	lp/lp
		25	1350 BC	Spain	Cueva Rubia-Valmayor/Madrid	D	Black	-	-	a/a	ov/ov	KM0/KM0	sb1/sb1	N/A	N/A	E/E	z/z	lp/lp

Table S1: continued.

		Sample	Age	Location	Excavation	Wild/ Domestic	Phenotype			Genotype								
Iron_Age	Asia	**BER2**	900 BC	Siberia	Om-1	D	Bay	-	-	A/A	ov/ov	KM0/KM0	sb1/sb1	C/C	N/A	E/e	z/z	lp/lp
		**OKG1**	400-300 BC	Mongolia	Olon-Kurin-Gol 10	D	Chestnut	-	-	A/a	ov/ov	KM0/KM0	sb1/sb1	C/C	N/A	e/e	z/z	lp/lp
		**OKG2**	400-300 BC	Mongolia	Olon-Kurin-Gol 10	D	Bay	Tobiano	-	A/a	ov/ov	KM1/KM0	sb1/sb1	C/C	N/A	E/E	z/z	lp/lp
		**OKG3**	400-300 BC	Mongolia	Olon-Kurin-Gol 10	D	Bay	-	Cream	A/a	ov/ov	KM0/KM0	sb1/sb1	C/cr	N/A	E/e	z/z	lp/lp
		**Bars1A**	370-150 BC	Siberia	Barsucij Log	D	Bay	Tobiano	-	A/a	ov/ov	KM1/KM0	sb1/sb1	C/C	N/A	E/e	z/z	lp/lp
		**Bars1B**	400-200 BC	Siberia	Barsucij Log	D	Bay	Tobiano	-	A/a	ov/ov	KM1/KM0	sb1/sb1	C/C	N/A	E/e	z/z	lp/lp
		**ARZ1-2**	800 BC	Sibiria	Arzan1	D	Chestnut	Leopard	-	A/a	ov/ov	KM0/KM0	sb1/sb1	C/C	N/A	e/e	z/z	LP/lp
		**ARZ1-3**	800 BC	Sibiria	Arzan1	D	Black	-	Silver	a/a	ov/ov	KM0/KM0	sb1/sb1	C/C	N/A	E/e	Z/z	lp/lp
		**ARZ2-1**	619-608 BC	Sibiria	Arzan2	D	Bay	-	-	A/a	ov/ov	KM0/KM0	sb1/sb1	C/C	N/A	E/E	z/z	lp/lp
		**ARZ2-2**	619-608 BC	Sibiria	Arzan2	D	Black	-	-	a/a	ov/ov	KM0/KM0	sb1/sb1	C/C	N/A	E/e	z/z	lp/lp
		**ARZ2-3**	619-608 BC	Sibiria	Arzan2	D	Chestnut	-	-	A/A	ov/ov	KM0/KM0	sb1/sb1	C/C	N/A	e/e	z/z	lp/lp
		**ARZ2-5**	619-608 BC	Sibiria	Arzan2	D	Chestnut	-	-	A/a	ov/ov	KM0/KM0	sb1/sb1	C/C	N/A	e/e	z/z	lp/lp
		**ARZ2-6**	619-608 BC	Sibiria	Arzan2	D	Bay	-	-	A/a	ov/ov	KM0/KM0	sb1/sb1	C/C	N/A	E/e	z/z	lp/lp
		**ARZ2-7**	619-608 BC	Sibiria	Arzan2	D	Black	-	-	a/a	ov/ov	KM0/KM0	sb1/sb1	C/C	N/A	E/e	z/z	lp/lp
		**ARZ2-8**	619-608 BC	Sibiria	Arzan2	D	Bay	-	Cream	A/a	ov/ov	KM0/KM0	sb1/sb1	C/cr	N/A	E/e	z/z	lp/lp
		**ARZ2-9**	619-608 BC	Sibiria	Arzan2	D	Bay	-	Cream	A/a	ov/ov	KM0/KM0	sb1/sb1	C/cr	N/A	E/e	z/z	lp/lp
		**ARZ2-10**	619-608 BC	Sibiria	Arzan2	D	Chestnut	Tobiano	-	A/a	ov/ov	KM1/KM0	sb1/sb1	C/C	N/A	e/e	z/z	lp/lp
		**ARZ2-12**	619-608 BC	Sibiria	Arzan2	D	Chestnut	-	-	A/a	ov/ov	KM0/KM0	sb1/sb1	C/C	N/A	e/e	z/z	lp/lp
		**ARZ2-13**	619-608 BC	Sibiria	Arzan2	D	Chestnut	-	-	A/a	ov/ov	KM0/KM0	sb1/sb1	C/C	N/A	e/e	z/z	lp/lp
		**ARZ2-14**	619-608 BC	Sibiria	Arzan2	D	Bay	-	-	A/A	ov/ov	KM0/KM0	sb1/sb1	C/C	N/A	E/e	z/z	lp/lp
		**Fen1**	cal. 905-800 BC	China	Fengtai	D	Bay	Tobiano	-	A/A	ov/ov	KM1/KM0	sb1/sb1	C/C	N/A	E/E	z/z	lp/lp
		**Fen2**	1000-800 BC	China	Fengtai	D	Bay	-	-	A/a	ov/ov	KM0/KM0	sb1/sb1	C/C	N/A	E/e	z/z	lp/lp
		**Fen3**	1000-800 BC	China	Fengtai	D	Bay	-	-	A/a	ov/ov	KM0/KM0	sb1/sb1	C/C	N/A	E/E	z/z	lp/lp
		**Fen4**	1000-800 BC	China	Fengtai	D	Bay	-	-	A/A	ov/ov	KM0/KM0	sb1/sb1	C/C	N/A	E/E	z/z	lp/lp
	Europe/ Asia(minor)	**Ois1**	900 BC – 300 AD	Belgium	Objectifs sud	D	Bay	-	-	A/A	N/A	KM0/KM0	N/A	N/A	PRL/PRL	E/E	N/A	lp/lp
		**Rid1**	800-600 BC	Estonia	Ridala (Saaremaa Island)	D	Chestnut	Tobiano	-	a/a	N/A	KM1/KM0	N/A	N/A	PRL/PRL	e/e	N/A	lp/lp
		**Rid2**	800-600 BC	Estonia	Ridala (Saaremaa Island)	D	Chestnut	Tobiano	-	a/a	N/A	KM1/KM0	N/A	N/A	PRL/PRL	e/e	N/A	lp/lp
		**P108**	2490 ± 70 BP	Denmark	Køge Bugt udfor Solrød	D	Chestnut	Tobiano	-	N/A	N/A	KM1/KM0	N/A	N/A	PRL/PRL	e/e	N/A	lp/lp
		**P098**	1-400 AD	Denmark	Hornborg	D	Bay OR Black	Leopard	-	N/A	N/A	KM0/KM0	N/A	C/C	PRL/PRL	E/e	N/A	LP/lp
	Iberia	**Vil1**	550-425 BC	Spain	Vilaris	D	Bay	-	-	A/a	ov/ov	KM0/KM0	sb1/sb1	C/C	PRL/PRL	E/E	z/z	lp/lp
		**4**	800 BC-6 AD	Spain	Soto de Medinilla -Valladolid	D	Black	-	-	a/a	ov/ov	KM0/KM0	sb1/sb1	C/C	N/A	E/E	z/z	lp/lp
Medieval	Europe/Asia(minor)	**P082**	1000-1300 AD	Denmark	Viborg Søndersø	D	Chestnut	-	-	A/a	N/A	KM0/KM0	N/A	N/A	PRL/PRL	e/e	N/A	lp/lp
		**Ote2**	600-1300 AD	Estonia	Otepää	D	Chestnut	-	-	A/a	N/A	KM0/KM0	N/A	C/C	PRL/PRL	e/e	N/A	lp/lp
		**Saa1**	600-1100 AD	Estonia	Saadjärve	D	Bay	Tobiano	-	A/A	N/A	KM1/KM0	N/A	N/A	PRL/PRL	E/e	N/A	lp/lp
		**Soo1**	900-1300 AD	Estonia	Soontagana	D	Chestnut	-	-	A/a	N/A	KM0/KM0	N/A	N/A	PRL/PRL	e/e	N/A	lp/lp
		**Dee47**	400-600 AD (Migration period)	Germany	Deersheim	D	Bay	-	(Pearl)	A/a	N/A	KM0/KM0	sb1/sb1	C/C	PRL/prl	E/e	z/z	lp/lp
		**Que40**	400-600 AD (Migration period)	Germany	Quedlinburg	D	Chestnut	Tobiano	-	N/A	N/A	KM1/KM0	sb1/sb1	C/C	PRL/PRL	e/e	z/z	lp/lp
		**Rat13**	400-600 AD (Migration period)	Germany	Rathewitz	D	Black	-	-	a/a	N/A	KM0/KM0	sb1/sb1	C/C	PRL/PRL	E/E	z/z	lp/lp
		**Zor3**	400-600 AD (Migration period)	Germany	Zorbau	D	Chestnut	-	-	a/a	N/A	KM0/KM0	N/A	C/C	PRL/PRL	e/e	z/z	lp/lp
		**Zwe2**	400-600 AD (Migration period)	Germany	Zwebendorf	D	Chestnut	-	-	a/a	N/A	N/A	sb1/sb1	C/C	PRL/PRL	e/e	z/z	lp/lp
		**FoEq001**	850-1050 AD	Iceland	Hrossbrein frá Garðsá 15223.	D	Bay	-	-	A/a	N/A	N/A	sb1/sb1	C/C	N/A	E/E	z/z	lp/lp
		**FoEq002**	850-1050 AD	Iceland	Hrossbrein frá Garðsá 15223.	D	Bay	-	-	A/a	ov/ov	KM0/KM0	sb1/sb1	C/C	PRL/PRL	E/e	z/z	lp/lp
		**FoEq003**	850-1050 AD	Iceland	Hrossbrein frá Garðsá 15223.	D	Black	-	-	a/a	ov/ov	KM0/KM0	sb1/sb1	C/C	PRL/PRL	E/e	z/z	lp/lp
		**FoEq008**	850-1050 AD	Iceland	Hrossabein frá Hrollaugsstöðum 15227.	D	Bay	-	Cream	A/a	ov/ov	KM0/KM0	sb1/sb1	C/cr	PRL/PRL	E/E	z/z	lp/lp
		**FoEq009**	850-1050 AD	Iceland	5856	D	Chestnut	-	-	N/A	ov/ov	KM0/KM0	sb1/sb1	C/C	PRL/PRL	e/e	z/z	lp/lp
		**FoEq023**	850-1050 AD	Iceland	6419 b. Granagil.	D	Chestnut	-	-	A/a	ov/ov	KM0/KM0	sb1/sb1	C/C	PRL/PRL	e/e	z/z	lp/lp
		**FoEq025**	850-1050 AD	Iceland	6948 Núpar hjá Laxamýri.	D	Chestnut	-	-	a/a	ov/ov	KM0/KM0	sb1/sb1	C/C	PRL/PRL	e/e	z/z	lp/lp
		**FoEq033**	850-1050 AD	Iceland	Kolsholt 11.10.1958, 1958:117	D	Chestnut	-	(Silver)	A/A	N/A	KM0/KM0	sb1/sb1	C/C	PRL/PRL	e/e	Z/z	lp/lp
		**FoEq034**	850-1050 AD	Iceland	Kolsholt 11.10.1958, 1958:117	D	Chestnut	-	(Silver)	N/A	ov/ov	KM0/KM0	sb1/sb1	C/C	PRL/PRL	e/e	Z/z	lp/lp
		**FoEq042**	850-1050 AD	Iceland	Hrossbein. Ytra-Garðshorn. 22.8.1958	D	Bay	-	-	A/a	ov/ov	KM0/KM0	sb1/sb1	C/C	PRL/PRL	E/E	z/z	lp/lp
		**FoEq044**	850-1050 AD	Iceland	Hrossbein. Ytra-Garðshorn. 22.8.1958	D	Chestnut	-	-	A/a	ov/ov	KM0/KM0	sb1/sb1	C/C	PRL/PRL	e/e	z/z	lp/lp
		**FoEq047**	850-1050 AD	Iceland	Hrossbein. Ytra-Garðshorn. 22.8.1958	D	Bay	-	-	A/a	ov/ov	KM0/KM0	sb1/sb1	C/C	PRL/PRL	E/e	z/z	lp/lp
		**FoEq052**	850-1050 AD	Iceland	Brandsstaðir í Blönduhlíð 1967-81.	D	Chestnut	-	-	N/A	N/A	N/A	N/A	C/C	PRL/PRL	e/e	z/z	lp/lp
		**FoEq060**	850-1050 AD	Iceland	11338A Hemla	D	Chestnut	-	-	N/A	N/A	N/A	N/A	N/A	PRL/PRL	e/e	z/z	lp/lp
		**FoEq065**	850-1050 AD	Iceland	11338A Hemla	D	Chestnut	-	-	N/A	N/A	N/A	sb1/sb1	N/A	N/A	e/e	z/z	lp/lp
		**FoEq066**	850-1050 AD	Iceland	Mörk #15 Landmannahreppur	D	Chestnut	-	-	N/A	N/A	N/A	N/A	N/A	PRL/PRL	e/e	z/z	lp/lp
		**FoEq068**	850-1050 AD	Iceland	Ytri-Neslönd 1960-46	D	Black	-	-	a/a	N/A	KM0/KM0	sb1/sb1	C/C	PRL/PRL	E/e	z/z	lp/lp
		**FoEq071**	850-1050 AD	Iceland	Eyrarteigur 1995-358 (Þórisárkuml)	D	Chestnut	-	-	N/A	N/A	N/A	sb1/sb1	N/A	PRL/PRL	e/e	z/z	lp/lp

Table S1: continued.

		Sample	Age	Location	Excavation	Wild/ Domestic	Phenotype			Genotype									
Medieval	Europe/Asia (minor)	**FoEq077**	850-1050 AD	Iceland	Miðsandfell 1982-57	D	Bay OR Black	-	-	*A/a*	*N/A*	*KM0/KM0*	*sb1/sb1*	*C/C*	*PRL/PRL*	*E/e*	*z/z*	*lp/lp*
		**Seb037**	700-800 AD	Slovakia	Sebastovce	D	Black	-	(Pearl)	*a/a*	*N/A*	*KM0/KM0*	*sb1/sb1*	*C/C*	*PRL/prl*	*E/e*	*z/z*	*lp/lp*
		**Seb048**	700-800 AD	Slovakia	Sebastovce	D	Bay	-	-	*A/a*	*ov/ov*	*KM0/KM0*	*sb1/sb1*	*C/C*	*PRL/PRL*	*E/e*	*z/z*	*lp/lp*
		**Seb052**	700-800 AD	Slovakia	Sebastovce	D	Bay	Tobiano + Leopard	-	*A/a*	*ov/ov*	*KM1/KM0*	*sb1/sb1*	*C/C*	*PRL/PRL*	*E/e*	*z/z*	*LP/lp*
		**Seb058**	700-800 AD	Slovakia	Sebastovce	D	Bay	Tobiano	-	*A/a*	*ov/ov*	*KM1/KM0*	*sb1/sb1*	*C/C*	*PRL/PRL*	*E/e*	*z/z*	*lp/lp*
		**Seb067**	700-800 AD	Slovakia	Sebastovce	D	Bay OR Black	-	-	*N/A*	*N/A*	*N/A*	*sb1/sb1*	*C/C*	*PRL/PRL*	*E/E*	*z/z*	*lp/lp*
		**Seb080**	700-800 AD	Slovakia	Sebastovce	D	Chestnut	Leopard	-	*A/a*	*ov/ov*	*KM0/KM0*	*sb1/sb1*	*C/C*	*PRL/PRL*	*e/e*	*z/z*	*LP/lp*
		**Seb093**	700-800 AD	Slovakia	Sebastovce	D	Black	-	-	*a/a*	*ov/ov*	*KM0/KM0*	*sb1/sb1*	*C/C*	*PRL/PRL*	*E/e*	*z/z*	*lp/lp*
		**Seb094**	700-800 AD	Slovakia	Sebastovce	D	Chestnut	-	-	*A/a*	*N/A*	*N/A*	*sb1/sb1*	*C/C*	*PRL/PRL*	*e/e*	*z/z*	*lp/lp*
		**Seb099**	700-800 AD	Slovakia	Sebastovce	D	Bay	-	-	*A/a*	*ov/ov*	*KM0/KM0*	*sb1/sb1*	*C/C*	*PRL/PRL*	*E/e*	*z/z*	*lp/lp*
		**Seb131**	700-800 AD	Slovakia	Sebastovce	D	Chestnut	-	-	*A/a*	*ov/ov*	*KM0/KM0*	*sb1/sb1*	*C/C*	*PRL/PRL*	*e/e*	*z/z*	*lp/lp*
		**Seb161**	700-800 AD	Slovakia	Sebastovce	D	Chestnut	-	-	*A/a*	*ov/ov*	*KM0/KM0*	*sb1/sb1*	*C/C*	*PRL/PRL*	*e/e*	*z/z*	*lp/lp*
		**Seb230**	700-800 AD	Slovakia	Sebastovce	D	Chestnut	-	-	*a/a*	*N/A*	*KM0/KM0*	*sb1/sb1*	*C/C*	*PRL/PRL*	*e/e*	*z/z*	*lp/lp*
		**Seb233**	700-800 AD	Slovakia	Sebastovce	D	Chestnut	-	-	*A/a*	*N/A*	*N/A*	*sb1/sb1*	*C/C*	*PRL/PRL*	*e/e*	*z/z*	*lp/lp*
		**Seb238**	700-800 AD	Slovakia	Sebastovce	D	Black	-	Silver	*a/a*	*N/A*	*KM0/KM0*	*sb1/sb1*	*C/C*	*PRL/PRL*	*E/E*	*Z/z*	*lp/lp*
		**Upps01**	600-800 AD	Sweden	Uppsala	D	Bay	-	-	*A/a*	*N/A*	*KM0/KM0*	*sb1/sb1*	*N/A*	*PRL/PRL*	*E/E*	*z/z*	*lp/lp*
		**Upps02**	600-800 AD	Sweden	Uppsala	D	Bay OR Chestnut	Leopard	-	*A/A*	*N/A*	*KM0/KM0*	*sb1/sb1*	*N/A*	*PRL/PRL*	*N/A*	*z/z*	*LP/lp*
		**Upps03**	600-800 AD	Sweden	Uppsala	D	Chestnut	-	-	*a/a*	*N/A*	*KM0/KM0*	*sb1/sb1*	*N/A*	*PRL/PRL*	*e/e*	*z/z*	*lp/lp*
		**Upps04**	680-890 AD	Sweden	Uppsala	D	Bay	-	-	*A/A*	*N/A*	*KM0/KM0*	*sb1/sb1*	*N/A*	*PRL/PRL*	*E/E*	*z/z*	*lp/lp*
		**Upps05**	1000-1150 AD	Sweden	Uppsala	D	Chestnut	-	-	*A/a*	*N/A*	*KM0/KM0*	*sb1/sb1*	*N/A*	*PRL/PRL*	*e/e*	*z/z*	*lp/lp*
		**Upps06**	800-1000 AD	Sweden	Uppsala	D	Bay	-	-	*A/A*	*N/A*	*KM0/KM0*	*sb1/sb1*	*N/A*	*PRL/PRL*	*E/E*	*z/z*	*lp/lp*
		**Upps07**	870-1160 AD	Sweden	Uppsala	D	Chestnut	-	-	*a/a*	*N/A*	*KM0/KM0*	*sb1/sb1*	*N/A*	*PRL/PRL*	*e/e*	*z/z*	*lp/lp*
		**Upps08**	1000-1200 AD	Sweden	Uppsala	D	Bay	-	-	*A/a*	*N/A*	*KM0/KM0*	*sb1/sb1*	*N/A*	*PRL/PRL*	*E/e*	*z/z*	*lp/lp*
		**Upps09**	800-900 AD	Sweden	Uppsala	D	Black	-	-	*a/a*	*N/A*	*KM0/KM0*	*sb1/sb1*	*N/A*	*PRL/PRL*	*E/E*	*z/z*	*lp/lp*
		**Upps10**	800-1050 AD	Sweden	Uppsala	D	Black	-	-	*a/a*	*N/A*	*KM0/KM0*	*sb1/sb1*	*N/A*	*PRL/PRL*	*E/e*	*z/z*	*lp/lp*
	Iberia	**Buz1**	500-600 AD	Spain	Buzanca 2 (Madrid)	D	Bay	-	(Pearl)	*A/A*	*N/A*	*KM0/KM0*	*sb1/sb1*	*C/C*	*PRL/prl*	*E/e*	*z/z*	*lp/lp*
		**29**	cal. 660-780 AD	Spain	Mucientes-Valladolid	D	Chestnut	-	-	*a/a*	*N/A*	*KM0/KM0*	*sb1/sb1*	*C/C*	*N/A*	*e/e*	*z/z*	*lp/lp*

Table S2: Primers used for the coat color SNPs amplification and Accession numbers of respective genes.

Locus/ Gene	Primer name	Primer sequence 5'-3'	Primer length	Accession number
KIT	Pa-KIT-13_F	CGTCATGACTCATTCGTGAGAA	22	AY048669
	Pa-KIT-13_R	GCTCTGAAGGTAACAAGCAACTAA	24	
KIT	Pa-KIT-16_F	TTTAAATGGCTTTCTTTTCTCC	22	AY874542
	Pa-KIT-16_R	TGCCAAGTCCCTATGAATACAC	22	
MATP	Pa-MATP_F	GCCATAACCATCACCATGATAG	22	AY187093
	Pa-MATP_R	GGCCCATCAATGAAGTCAG	19	
MATP	Pa-Pearl_F	ACTACCGCTACCTTTGCATCAG	22	
	Pa-Pearl_R	TGAAATCTGTGAAGAAGAGCAT	22	
MC1R	Pa-MC1R_F	GCACTCACCCATGTACTACTTCAT	24	X98012
	Pa-MC1R_R	GCACGTTGCTCATGCTCAC	19	
PMEL	Pa-SILV_F	TCCTTCTTCTTCTCCCAAATCA	22	DQ665301
	Pa-SILV_R	GAGCTGAGCCCTGCTTCATAA	21	
ASIP	Pa-ASIP_F	CAAGAAATCCAAAAAGATCAGC	22	AF288358
	Pa-ASIP_R	ATGAGAAGTCCAAGGCCTACCT	22	
TRPM1	Pa-LP-rev_F	GAGCTGCTGAACTGGGTATGT	21	XM_001492235
	Pa-LP-rev_R	TCTCCATGATCCCAAGCAAT	20	
TRPM1	Pa-LP-for_F	AGATCGAGCTGCTGAACTGG	20	XM_001492235
	Pa-LP-for_R	TCTCCATGATCCCAAGCAAT	20	

Table S3: Allele frequencies in the observed sample over the time periods employed for the simulations analysis. The frequencies are slightly different than in the subsets employed in the temporal test of allele frequencies.

**Time periods**	**Observed frequencies**							
	ASIP	KIT13-Tobiano	KIT16-Sabino	MATP-Cream	MATP-Pearl	MC1R	PEML17-SILV	TRPM1-LP
**Allele**	*A*	*KM0*	*sb1*	*C*	*PRL*	*E*	*z*	*lp*
**Ple**	0.967	1.000	1.000	1.000	NA	1.000	1.000	0.867
**MNe**	0.375	1.000	1.000	1.000	1.000	1.000	1.000	1.000
**CoA**	0.325	0.948	0.964	1.000	1.000	0.646	1.000	0.875
**EBA**	0.270	1.000	1.000	1.000	1.000	0.889	1.000	0.816
**MBA**	0.476	1.000	1.000	1.000	1.000	0.609	1.000	0.964
**LBA**	0.431	0.950	0.933	1.000	1.000	0.733	1.000	0.967
**EIA**	0.522	0.896	1.000	0.952	1.000	0.396	0.976	0.979
**IrA**	0.500	0.813	1.000	0.929	1.000	0.625	1.000	0.938
**Med**	0.402	0.956	1.000	0.990	0.972	0.345	0.971	0.973

Table S4: Number of individuals for each detected phenotype per period and region. The last two rows summarize the number of phenotypes of the different regions per period as well as all phenotypes of this time period, respectively.

Period			Plesitocene/Mesolithic (> 4000 BC)			Neolithic/Copper Age (4000-2700 BC)			Early Bronze Age (2100-1600 BC)			Mid/Late Bronze Age (1600-900 BC)			Iron Age (900 BC – 400 AD)			Middle Ages (< 400AD)		
Sample size			N = 28			N = 29			N = 28			N = 31			N = 31			54		
Region			Asia	Europe/ Asia(minor)	Iberia	Asia	Europe/ Asia(minor)	Iberia	Asia	Europe/ Asia(minor)	Iberia	Asia	Europe/ Asia(minor)	Iberia	Asia	Europe/ Asia(minor)	Iberia	Asia	Europe/ Asia(minor)	Iberia
Basic	Pattern	Dilution																		
Bay	-	-	5	9-10	4	9	4-6		4	6-8	1	5	3	3	7	1	1		10-13	1
Bay	Leopard	-		5		2	2			4		1				1			0-1	
Bay	Sabino + Leopard	-				2														
Bay	Tobiano	-				1	1						1		4				2	
Bay	Sabino	-										2	1							
Bay	Tobiano + Leopard	-																	1	
Bay	-	Cream													3				1	
Black	-	-		2-3	2	1	3-5		2	3-5	2	3	4	2	2		1		6-9	
Black	Leopard	-								3										
Black	-	Silver													1				1	
Chestnut	-	-				2				1		1	2		5				24	1
Chestnut	Tobiano	-										2			1	3			1	
Chestnut	Sabino	-											1							
Chestnut	Leopard	-													1				1-2	
Number of horses with spotted/diluted coat color			5			8			7			8			14			8		
Number of horses with basic coat color			23			21			21			23			17			46		
Number of phenotypes			1	3	2	6	4		2	5	2	6	6	2	8	3	2		10-11	2
Number of phenotypes for all regions/period			3			6			5			8			9			10-11		

Table S5: *P*-values for the temporal test of allele frequencies applied to pairs of consecutive subsets of samples with similar ages in the nine time periods analyzed in this study. NA refers to a test that was not applied because the frequencies stayed the same between the periods. The grouping of samples is shown in figure SU. The numbers separated by a diagonal are the counts of alleles (in the order as they appear in the first row) corresponding to the first temporal sample of the comparison; and the numbers below are the *p*-values of the temporal test for N_e of 10^3, 10^4 and 10^5, respectively.

**Comparison**	**Average age (1st sample)**	**Nr. of generations**	**ASIP**	**KIT13-Tobiano**	**KIT16-Sabino**	**MATP-Cream**	**MATP-Pearl**	**MC1R**	**PEML17-SILV**
			A/a	*KM0/KM1*	*sb1/SB1*	*C/cr*	*prl/Prl*	*E/e*	*z/Z*
**Ple vs MNe**	17146	2023	21/1	22/0	22/0	22/0	-/-	20/0	22/0
			0.0717	-	-	-	-	-	-
			**0.0011**	-	-	-	-	-	-
			**<0.0001**	-	-	-	-	-	-
**MNe vs CoA**	7029	311	4/10	14/0	14/0	14/0	2/0	14/0	14/0
			0.7840	0.7616	0.8637	-	-	0.0572	-
			0.6801	0.4217	0.6180	-	-	**0.0065**	-
			0.6607	0.3613	0.5582	-	-	**0.0035**	-
**CoA vs EBA**	5472	206	12/22	45/3	44/2	46/0	2/0	22/16	48/0
			0.7599	0.4083	0.4871	-	-	0.0185	-
			0.6537	0.2965	0.3545	-	-	**0.0009**	-
			0.6347	0.2717	0.3204	-	-	**0.0003**	-
**EBA vs MBA**	4442	105	7/17	24/0	24/0	24/0	4/0	24/0	24/0
			0.3130	-	-	-	-	**0.0088**	-
			0.2190	-	-	-	-	**0.0016**	-
			0.2071	-	-	-	-	**0.0012**	-
**MBA vs LBA**	3916	113	10/11	28/0	28/0	28/0	8/0	14/9	28/0
			0.9472	0.4027	0.3555	-	-	0.2149	-
			0.9337	0.2873	0.2145	-	-	0.2316	-
			0.9314	0.2700	0.1913	-	-	0.3617	-
**LBA vs EIA**	3352	146	25/29	53/3	52/4	48/0	2/0	42/14	56/0
			0.8617	0.3347	0.3186	0.1732	-	**0.0027**	-
			0.8013	0.2091	0.2109	0.1049	-	**<0.0001**	-
			0.7918	0.1861	0.1926	0.0908	-	**<0.0001**	-
**EIA vs IrA**	2624	59	12/16	26/4	24/0	22/2	6/0	8/22	24/0
			0.7711	0.5391	-	1.0000	-	0.0317	-
			0.7472	0.5019	-	1.0000	-	**0.0183**	-
			0.7425	0.5004	-	1.0000	-	**0.0166**	-
**IrA vs Med**	2330	239	7/7	11/3	12/0	11/1	2/0	9/5	12/0
			0.7083	0.1217	-	0.6628	1.0000	0.1767	0.9925
			0.5813	**0.0290**	-	0.2832	1.0000	0.0486	0.9969
			0.5573	**0.0218**	-	0.2273	1.0000	0.0340	0.9988
**(Med)**	1134		34/48	77/3	86/0	63/1	93/1	34/64	91/1

Table S6. *P*-values of comparisons of observed allele frequencies among contemporary locations. As obtained by means of a contingency table Chi-square test.

**Time bin**	**Locus (alleles ancestral/derived)**	**Compared locations (# of alleles, ancestral/derived)**	***P*-value**
**4000-3000 y BC**	ASIP (A/a)	Germany (4/4); Siberia (7/3); Ukraine (6/7)	0.049
**3000-2000 y BC**		Turkey (5/15); Siberia (4/4)	0.204
**600-1200 y AD**		Estonia (4/2); Iceland (10/14); Slovakia; (9/17); Sweden (9/11)	0.542
**600-1200 y AD**	MC1R (E/e)	Estonia (4/2); Iceland (11/27); Slovakia (10/18); Sweden (10/8)	0.187
**600-1200 y AD**	TRPM1 (lp/LP)	Estonia (6/0); Iceland (38/0); Slovakia (28/2); Sweden (19/1)	0.419

Table S7. Priors and setting of the MCMC procedure employed for the final inference of selection coefficients and other parameters. *Standard deviation of the jump distribution. % indicates that the S.D. corresponds to the indicated percentage of the available parameter range. **The bounds of the time to the introduction of alleles were variable, and set between the oldest sample and the first appearance of the allele.

Setting/Operator	Value	Type of distribution
**Chains run**	20	-
**Simulations per chain**	20000	-
**Interval for posterior probability storing**	100	-
**Probability of being introduced**	0.5	-
**S.D. generation time***	0.1	Normal
**S.D. N_{e1}***	5%	Normal
**S.D. N_{e2}***	5%	Normal
**S.D. Initial allele frequency***	2.5%	Normal
**S.D. Time to the allele introduction***	2.5%	Normal
**S.D. Selection coefficients***	0.25%	Normal

Prior distributions	Probability distribution	Parameters
**Selection Coefficients**	Exponential	[0.1]
**N_{e1}* (initial population size)**	Uniform	[100,100000]
**N_{e2}* (final population size)**	Uniform	[N_{e1},1000000]
**Generation time**	Normal	M=5.0, σ=0.5
**Initial allele frequency**	Uniform	[0.0,1.0]
**Time to the introduction of the allele**	Uniform	[Min, Max]**
**Time to the start of the demographic growth**	Normal	[5600yBP,100]

References

1 Schraiber, J. G., Evans, S. N. & Slatkin, M. Bayesian Inference of Natural Selection from Allele Frequency Time Series. *Genetics* **203**, 493-511, doi:10.1534/genetics.116.187278 (2016).

2 Bollback, J. P., York, T. L. & Nielsen, R. Estimation of 2Nes from temporal allele frequency data. *Genetics* **179**, 497-502, doi:10.1534/genetics.107.085019 (2008).

3 Malaspinas, A. S., Malaspinas, O., Evans, S. N. & Slatkin, M. Estimating allele age and selection coefficient from time-serial data. *Genetics* **192**, 599-607, doi:10.1534/genetics.112.140939 (2012).

4 Steinrücken, M., Bhaskar, A. & Song, Y. S. A novel spectral method for inferring general diploid selection from time series genetic data. *The Annals of Applied Statistics* **8**, 2203-2222, doi:10.1214/14-aoas764 (2014).

5 Mathieson, I. & McVean, G. Estimating selection coefficients in spatially structured populations from time series data of allele frequencies. *Genetics* **193**, 973-984, doi:10.1534/genetics.112.147611 (2013).

6 Ronquist, F. *et al.* MrBayes 3.2: efficient Bayesian phylogenetic inference and model choice across a large model space. *Syst Biol* **61**, 539-542, doi:10.1093/sysbio/sys029 (2012).

7 Sandoval-Castellanos, E. Testing temporal changes in allele frequencies: a simulation approach. *Genet Res (Camb)* **92**, 309-320, doi:10.1017/S0016672310000339 (2010).

8 Ludwig, A. *et al.* Twenty-five thousand years of fluctuating selection on leopard complex spotting and congenital night blindness in horses. *Philos Trans R Soc Lond B Biol Sci* **370**, 20130386, doi:10.1098/rstb.2013.0386 (2015).

9 Nielsen, R. & Wakeley, J. Distinguishing Migration From Isolation: A Markov Chain Monte Carlo Approach. *Genetics* **158**, 885-896 (2001).

10 Wutke, S. *et al.* The Origin of Ambling Horses. *Current Biology* **26**, R1-R3, doi:10.1016/j.cub.2016.07.001 (2016).
